# Exploring the temporal dynamics of methane ebullition in a subtropical freshwater reservoir

**DOI:** 10.1371/journal.pone.0298186

**Published:** 2024-03-27

**Authors:** Lediane Marcon, Tobias Bleninger, Michael Männich, Mayra Ishikawa, Stephan Hilgert, Andreas Lorke

**Affiliations:** 1 Post-graduate Program on Water Resources and Environmental Engineering (PPGERHA), Federal University of Paraná, Curitiba, Brazil; 2 Institute for Environmental Sciences, RPTU Kaiserslautern-Landau, Landau, Germany; 3 Department of Environmental Engineering, Federal University of Paraná, Curitiba, Brazil; 4 Institute for Water and River Basin Management, Department of Aquatic Environmental Engineering, Karlsruhe Institute of Technology, Karlsruhe, Germany; University 20 Aout 1955 skikda, Algeria, ALGERIA

## Abstract

The transport of methane from sediments to the atmosphere by rising gas bubbles (ebullition) can be the dominant, yet highly variable emission pathway from shallow aquatic ecosystems. Ebullition fluxes have been reported to vary in space and time, as methane production, accumulation, and bubble release from the sediment matrix is affected by several physical and bio-geochemical processes acting at different timescales. Time-series analysis and empirical models have been used for investigating the temporal dynamics of ebullition and its controls. In this study, we analyzed the factors governing the temporal dynamics of ebullition and evaluated the application of empirical models to reproduce these dynamics across different timescales and across different aquatic systems. The analysis is based on continuous high frequency measurements of ebullition fluxes and environmental variables in a mesotrophic subtropical and polymictic freshwater reservoir. The synchronization of ebullition events across different monitoring sites, and the extent to which ebullition was correlated to environmental variables varied throughout the three years of observations and were affected by thermal stratification in the reservoir. Empirical models developed for other aquatic systems could reproduce a limited fraction of the variability in observed ebullition fluxes (R^2^ < 0.3), however the predictions could be improved by considering additional environmental variables. The model performance depended on the timescale. For daily and weekly time intervals, a generalized additive model could reproduce 70 and 96% of ebullition variability but could not resolve hourly flux variations (R^2^ = 0.19). Lastly, we discuss the potential application of empirical models for filling gaps in ebullition measurements and for reproducing the main temporal dynamics of the fluxes. The results provide crucial information for emission estimates, and for the development and implementation of strategies targeting at a reduction of methane emissions from inland waters.

## Introduction

Freshwater reservoirs play a vital role in the global carbon cycle [1] and emit large amounts of greenhouse gases, including methane, to the atmosphere [2]. Bubble mediated transport of methane, i.e. methane ebullition, is an important pathway of methane emissions in shallow lentic ecosystems [3]. At the same time, ebullition is an episodic and highly variable process [4, 5].

Spatially, ebullition is reported to vary across different water bodies as well as within individual systems. Sediment deposition, quality and quantity of organic matter content, nutrients, and sediment temperature have been reported to affect methane production in bottom sediments [6–8]. The accumulation of methane, the formation of gas voids, and the release of gas bubbles from the sediment matrix is controlled by the complex interplay of physical aspects, including grain size, total pressure and pressure changes at the sediment-water interface [9, 10] and biochemical conditions, such as methane oxidation [11], or zooplankton migration [12].

In time, ebullition is highly variable at timescales ranging from minutes to seasons [13], as it is influenced by the dynamics of multiple environmental variables. Atmospheric conditions of low pressure and rapid decrease of hydrostatic pressure are known to promote bubble release from the sediment, as with reduced pressure the bubbles expand and gain buoyancy [14–16]. Other environmental variables, such as strong wind and high current velocities [5, 17], and warmer temperatures [18] are also reported to enhance ebullition fluxes.

The overlapping and sometimes delayed (asynchronous) effects of the environmental variables on ebullition, make it challenging to predict the fluxes. Past studies proposed mechanistic approaches and empirical relationships to predict ebullition fluxes from aquatic systems. In mechanistic approaches, ebullition is simulated by resolving the methane transport-reaction equations with ebullition occurring above a threshold, commonly based on pressure, dissolved gas concentration, or volume of free gas [19–21]. The process-based models have been developed in different ways, however, they commonly require information on methane production and loss (others than ebullition) from the sediment [22, 23], while more complex models require additional information on sediment properties, such as porosity and effective stress [20, 24]. The required input data (e.g., boundary conditions), and the availability of measurements for calibration make the application of mechanistic models for the simulation of ebullition challenging.

Statistical and data-driven models are less demanding in terms of input variables, however the quality and quantity of input data directly affect the model performance. These empirical models have been applied in previous studies for both testing the dependency of ebullition on diverse environmental variables and for estimating the ebullition flux, and considered temperature [18, 25], chlorophyll-a [26], littoral area and radiance [27], sediment organic matter content [28], wind speed [29], pressure, and pressure changes [29–31]. Nevertheless, the applicability of these statistical and data-driven models across diverse systems and timescales for reproducing ebullition dynamics has rarely been addressed. Ebullition drivers were shown to change from one location to another of the same reservoir [29], while iterative forecasting models with continuous update of model coefficients can potentially improve the prediction of ebullition dynamics at weekly time scales [32]. Additionally, a recent study [33] highlighted that advances are needed for the application of empirical models for the estimation of greenhouse gas emissions from inland waters.

The analysis of ebullition time-series provide important and useful insights into ebullition dynamics [13], while the use of ebullition time series in combination with time-series of environmental variables allows for the identification of main environmental drivers [29, 30]. At the same time, empirical models are powerful tools to fill measurement gaps, to support estimates of emissions from systems where mechanistic approaches are no available, and to potentially reproduce the short-term temporal dynamics of ebullition. The ability of predicting short-term temporal dynamics of ebullition, in contrast to mean fluxes, is important to identify patterns and trends that may have been missed by considering mean fluxes. Moreover, as ebullition is a highly dynamic process that can vary greatly at short timescales [13, 16], predicting mean fluxes alone may not accurately capture this variability. Lastly, the identification of hot moments in ebullition fluxes can guide practical applications targeting a reduction of methane emissions from freshwater reservoirs [34, 35].

In the present study, we aim to improve the understanding of the temporal dynamics of ebullition and its main controls. We revisited and complemented high frequency ebullition measurements in a polymictic mesotrophic subtropical freshwater reservoir with an integrated measurement approach. We analysed ebullition time-series in combination with an extensive high temporal resolution dataset of environmental variables with the objectives (1) to evaluate the dependency of ebullition on different drivers over time; (2) to test to what extent empirical models can be applied across different systems to reproduce ebullition temporal dynamics; and (3) to verify the capabilities of empirical models to reproduce ebullition temporal dynamics at different timescales.

## Material and methods

### Study site

**Fig 1:**
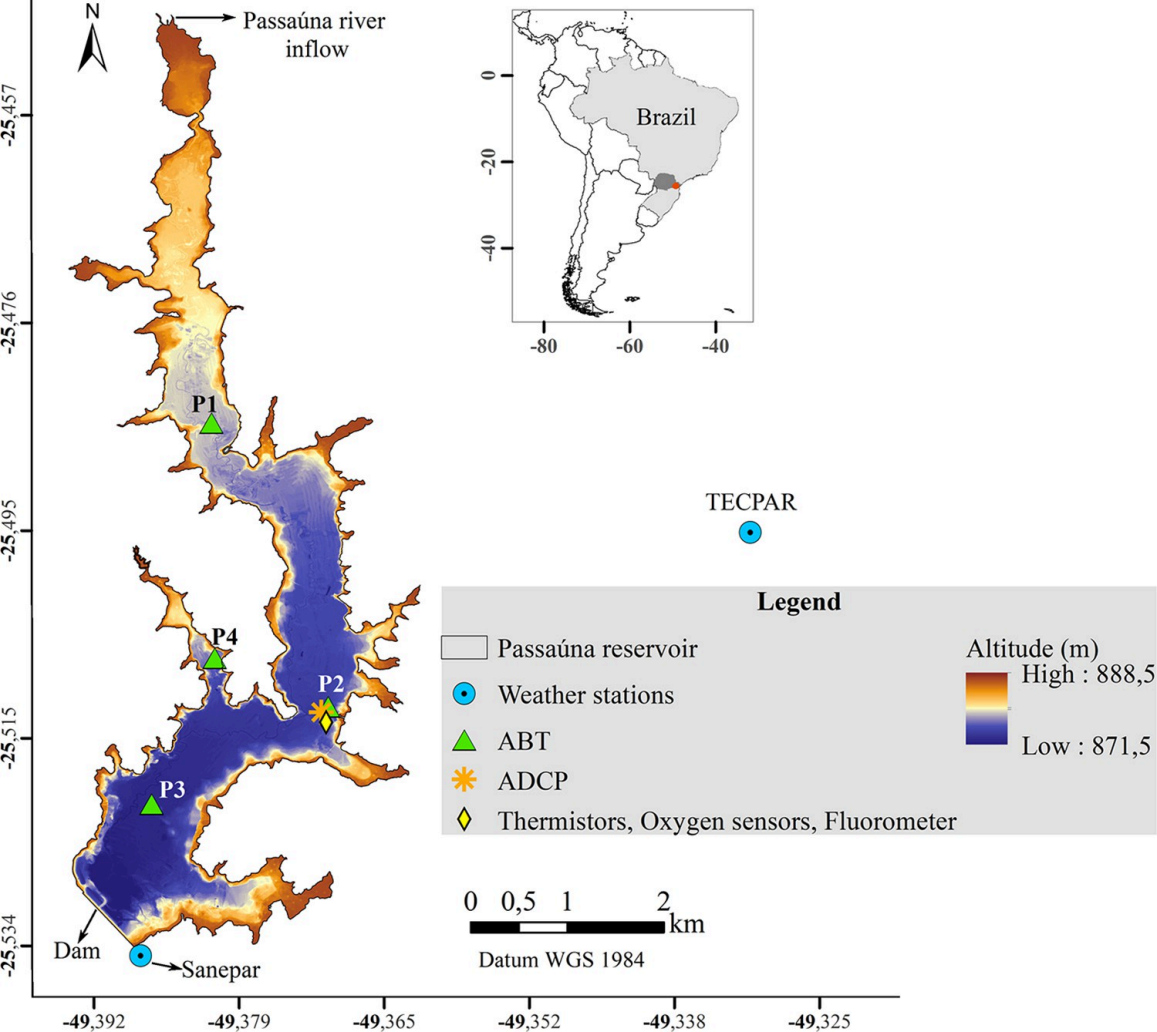
Bathymetric map of Pasaúna Reservoir with the location of sampling sites. The inset map shows the location of the reservoir in Brazil and South America. The location of sensors in the reservoir is marked by different symbols (see legend) and the colour scale shows the reservoir bathymetry. Reservoir’s bathymetric data and boundaries are republished from [38] under a CC BY license, with permission from ELSEVIER, original copyright 2021.The measurement period of each sensor is provided in S2 Fig. Ebullition was monitored at 4 sites using automated bubble traps (ABT). The shallowest (~8 m depth) upstream site was labelled as P1 (also referred as Park, as there is a natural park at the left-hand reservoir bank), site P2 (Intake) at ~12 m depth was placed in front of the water intake facility, site P3 (Dam) is at the deepest (~14 m) region of the reservoir, and site P4 (Arm) was placed in a side-arm of the reservoir which has ~10 m depth.

We analyzed measurements from a subtropical drinking-water reservoir (Passaúna Reservoir), located in the southern part of Brazil (25.53°S and 49.39°W, Fig 1). The reservoir was created in 1989 by a dam constructed in the Passaúna River. It has a surface area of 8.5 km^2^, an average water depth of 8.3 m, and maximum depth of ~18 m near the dam. The main water inflow to the Reservoir is the Passaúna River (average of 1.7 m^3^ s ^– 1^ [36]) and the main water outflows are the withdrawal at the water treatment plant (average of 1.8 m^3^ s ^– 1^), a bottom outlet near the dam (with continuous discharge of 0.5 m^3^ s ^– 1^), and a free overflow at the spillway [37].

Several environmental parameters were monitored in the reservoir from February 2017 to February 2020 and were reported in previous studies. The field monitoring was conducted in cooperation with the reservoir operator Sanepar (Sanitation Company of Paraná State), which granted access to the study site and provided relevant data. In years without severe droughts, the water level in the Reservoir fluctuates only slightly (for instance, during the monitoring period, the maximum variation was 1.4 m over 3 months from July to September 2018). Passaúna is a polymictic mesotrophic reservoir, the maximum observed water temperature near the sediment was of 23.2°C (in February 2019), whereas the coldest temperature of 16°C occurred in June 2018 and July 2019 [39]. During warmer periods, when the reservoir was thermally stratified, the bottom water layer can become anoxic with dissolved oxygen concentrations less than 1 mg L^−1^. High-frequency internal waves with periods ranging from 2.1 to 17.1 minutes were also detected in the reservoir, in which short periods (< 6.2 minutes) internal waves were reported during stratified conditions, whereas internal waves with longer periods were detected during mixed conditions [39].

The bottom sediment is characterized by unconsolidated fine-grained material with an average Loss on Ignition (LOI at 550°C as an indicator for organic matter content) of 17% and a highest fraction (up to 50%) found on the deepest regions [38]. Within the reservoir, two main locations are preferred for sediment accumulation, upstream near to the main river inflow and in the deepest region in front of the dam [38]. The top 10 cm of organic-rich sediment can produce and store methane. The average potential methane production for this top layer obtained from incubated sediments was 2.2 mg CH_4_ L^−1^ d^−1^, while the mean gas content stored in the sediment matrix was acoustically estimated as 4.6 L m^−2^ in 2019 [40].

### Measurements and data

Here we analyzed time series of ebullition fluxes in combination with additional data from previous studies, including dissolved oxygen concentration, water temperature, water level, flow velocity, acoustic backscatter in the water column, chlorophyll-a, and meteorological variables (solar radiation, wind velocity and direction, atmospheric pressure, air temperature, and humidity). All measurements and data are briefly described below and a summary of data availability throughout the analysis period is shown in supporting information S1 Fig (sensor locations are shown in Fig 1). Data gaps in the time series were caused by different issues, including sensor failure, battery shortage, and maintenance requirements. Monthly field campaigns were performed for data acquisition, sensor cleaning and maintenance.

#### Ebullition flux and potential methane production

Monitoring of ebullition fluxes in the reservoir started in February 2017 and the results from the first year of measurements were presented in [41]. Here we continued the gas flux monitoring with measurements until February 2020 at four locations (P1 to P4, see Fig 1) with automated bubble traps (ABT, Senect GmBH, Germany). The bubble traps were fixed to buoys and submerged in the water column. Anchors weights attached to secondary buoys were used to keep the ABT position at 1.7–2.7 m bellow the water surface (6 to 11 m above the bed). The ABT collected rising bubbles with an inverse canvas funnel of 1 m diameter (0.78 m^2^ opening area) attached to an aluminum frame. The gas was channeled into an acrylic tube where the volume was measured by a calibrated differential pressure sensor. The ABT recorded gas fill height, temperature, and pressure at 30 s time intervals in an internal data logger. The accumulated gas was flushed by a valve when the maximum fill height was reached, or every 12 hours.

The gas flux (in mL m^−2^ d^−1^) was calculated for defined time intervals (5 min, 10 min, 1 hr, and 1 d) as the total gas volume at standard pressure (1 atm) and temperature (20°C), as described in [41]. To obtain the methane ebullition flux (in mgCH_4_ m^−2^ d^−1^), the gas volume was converted to methane mass using the ideal gas law with in-situ temperature and pressure, and a mean constant methane fraction within the bubbles of 68.9% [40]. During the monitoring period, we recorded valid data for 605 d at location P1, 680 d at P2, 549 d at P3, and 274 d at P4.

Time series at 5 min, 10 min, 1 hr, and 1 d time intervals of the potential methane flux at the sediment water interface (PSWI) as a function of in-situ temperature were obtained from [40]. The potential fluxes were estimated from depth-integrated production rates measured in laboratory incubations of sediment cores sampled near the ABT’s locations, and corrected for water temperature at the respective sampling sites.

#### Velocity field and acoustic backscatter

Vertical profiles of flow velocity were adopted from [39]. The measurements were conducted using an upward-looking Acoustic Doppler Current Profiler (ADCP Signature 1000, Nortek AS, Norway), which was deployed from February 2018 to February 2019 at the reservoir bottom next to ABT P2 (Fig 1). Vertical profiles of the three components of the mean flow velocity were measured with 0.5 m vertical resolution over the entire water column and with a temporal resolution of 5 min. In addition, the ADCP recorded high resolution profiles (0.04 m vertical resolution and sampling frequency of 1 – 4 Hz) of the vertical velocity component (HR-Burst mode), and acoustic backscatter strength (full water column profile). The temporal variations in acoustic backscatter measured in Passaúna was previously used as an indicator of zooplankton migration in the water column [39]. The ADCP also recorded the pressure, which was used to calculate time series of water depth.

Here we used the velocity time series to calculate the variance and the magnitude of root-mean square fluctuations of the vector-averaged mean flow speed at the lowest sampling depth (1.2 m above the sediment) and of the high-frequency fluctuations in the vertical velocity (HR-Burst measurements for the water layer between 1 m to 3 m above the bed), respectively. The statistical properties were estimated for defined time intervals (mean horizontal current speed at 1 hr and 1 d and vertical velocity at 5 min, 10 min, 1 hr, and 1 d). The dissipation rate of turbulent kinetic energy near the bottom (0.64–0.68 m from the bottom) at 10 min time intervals was available from a previous study [39].

#### Dissolved oxygen concentration, water temperature, chlorophyll-a, and inflow discharge

Dissolved oxygen concentration (~1 m below the water surface and 2 m above the bed) and water temperature near the sediment were obtained from [39]. Dissolved oxygen was measured at 5 min time interval by optical sensors (miniDOT, Precision Measurement Engineering, Inc.) and the water temperature was measured by temperature loggers (Minilog-II-T, Vemco, Bedford, NS, Canada) at 1 min time intervals [39]. The difference in water temperature between the surface and the bottom were used to calculate the relative water column stability (RWCS), which represents the relative thermal resistance to mixing [42]:

$$RWCS=\frac{\rho_{bottom}-\rho_{surface}}{\rho_{4{}^{\circ}C}-\rho_{5{}^{\circ}C}}$$ (1)

*ρ_bottom_* and *ρ_surface_* are the water densities at the bottom and at the surface, respectively, *ρ*_4°*C*_ and *ρ*_5°*C*_ are the water densities at temperatures of 4°C and 5°C. The water density as a function of temperature was calculated using the UNESCO equation [43]. For RWCS > 56.5, the reservoir is considered as thermally stratified, RWCS < 16.3 indicates a mixed water column, and 16.3 < RWCS < 56.5 indicates partial stratification [44, 45]. In addition, the Schmidt stability (S_T_) was also used as an indicator for mixed or stratified conditions [39]. Here we assumed that the sediment temperature was in equilibrium with the overlaying water, and therefore we adopted the bottom water temperature as the sediment surface temperature.

Time series of daily averaged chlorophyll-a concentration near the water surface were adopted from [37] and were estimated from continuous measurements with a fluorometer (FluoroProbe III, bbe moldaenke GmbH, Germany) deployed at 1.4 m water depth at the monitoring site P2. The daily time-series of the Passaúna river inflow discharge was obtained from [46].

#### Meteorological data

Wind velocity and direction, solar radiation, air temperature, relative humidity, and atmospheric pressure were recorded by two weather stations located near and at the reservoir, respectively (see Fig 1 for locations). A station at the reservoir dam was installed in May 2018 (by the reservoir operator Sanepar) and recorded data at 10 min time intervals. A second station was located at the Technology Institute of Parana (TECPAR) ~4 km away from the reservoir and recorded data at 1 min time intervals. Both data sets had frequent gaps exceeding 10 days. Therefore, we combined the measured variables from both weather stations (except for wind direction) to obtain a single continuous data set, in which the data of the Sanepar station was complemented with the TECPAR measurements using linear fits between data from both stations. For our analysis, the meteorological data were averaged over time intervals of 5 min, 10 min, 1 hr, and 1 d.

### Data processing and analysis

#### Time series preparation

Several quality checks were applied to all time-series to remove spurious data (e.g. identify and exclude sensor failure and sensors readings during maintenance/deployment periods). The different environmental parameters were recorded at various discrete time intervals (e.g. 30 s, 1 min, or 10 min) with different starting and ending times. Therefore, after the data check and cleaning, time-series with regular and fixed time intervals (5 min, 10 min, 1 hr, 1 d) were created for each variable in which the time refers to the end of the considered intervals. Initially, high-resolution timetables (5 min and 10 min time steps) were created, and linear interpolation was used when necessary to calculate the values at the desired pre-defined timesteps (for instance, for adjusting the time-step from measured at 13:13 to pre-defined time at 13:15). In a second step, the 10 min time series were used to calculate hourly and daily timetables by averaging.

Some variables were originally monitored/calculated only at longer time-intervals (such as the velocity variance of horizontal currents, which were only available for time intervals > 1 hr, and Chlorophyll-a measurements were at daily time steps only). In these cases, we opted for not downscaling the values to shorter time intervals, as this could introduce artificial temporal dynamics and affect the subsequent analysis of the data. In total, daily time series of 30 environmental variables were obtained (excluding ebullition fluxes, all the variables are listed in S4 Fig). The final time series are available in the Zenodo repository [47].

#### Statistical analysis of ebullition time series

After data preparation, the temporal dynamics of environmental variables and ebullition fluxes were evaluated by basic statistics (averages ± standard deviations, median values, and boxplots). The normality of the time-series was checked using Kolmogorov-Smirnov tests. The coherence of ebullition fluxes at different monitoring sites and timescales was tested using Spearman rank correlations (rs, for significance level of 0.05) and the Kuramoto order parameter (r). As described by [48], the Kuramoto order parameter indicates synchrony among different oscillators, in which r ranges between 0 and 1, with r = 1 indicating perfect synchrony (oscillations in phase) and r = 0 perfect asynchrony (oscillations with opposing phase). Because the calculation of the Kuramoto order parameter requires continuous time series, we used daily ebullition time-series simultaneously recorded at study sites P1, P2, and P3 for two periods: December 13^th^ 2018 –April 24^th^ 2019 and June 05^th^ 2019 –October 16^th^ 2019. Location P4 was not included due to gaps in the measurements. For the Kuramoto order parameter, we first filled missing values by linear interpolation (for gaps up to 8 data points), removed linear trends in the time-series, normalized them using z-scores, and finally calculated r for each time step based on the instantaneous phase of each oscillator in a complex plane [48, 49]. Therefore, the calculated r represents the synchrony of daily ebullition among the monitoring sites P1, P2, and P3.

The frequency distribution of variance in the ebullition time-series (linearly detrended 5 min time-series) was analyzed using power spectra. The spectra were estimated using the Welch’s method with a Hamming window and 50% overlap. Wavelet analysis was used to identify the variability of any existing periodicity over time. For this, the analytic Morse wavelet was used to obtain continuous wavelet transforms. Additionally, coherence between ebullition and its main drivers in time-frequency domain was analysed using wavelet coherence analysis. This analysis was conducted using the analytical Morlet wavelet in MatLab (MathWorks R2023a). Alongside the magnitude-squared wavelet coherence, the phase lag of ebullition with respect to the driver was also obtained. The phase lag was graphically represented by arrows, with the arrow direction corresponding to the phase lag on the unit circle.

#### Ebullition triggers and prediction

The relationship between methane ebullition flux and possible environmental drivers (or controls) was initially explored using Spearman rank correlations (for significance level of 5%) and Principal Component Analysis (PCA) with the main variables with significant correlation and grouped by reservoir mixing conditions (based on RWCS described above). The interrelation between methane ebullition and main (single) drivers was further evaluated with wavelet coherence analysis, which allows to evaluate when and at what frequencies the two time-series were correlated. The method is based on continuous wavelet transforms to calculate the wavelet cross-spectrum and to obtain the magnitude-squared coherence between two time-series. Values of magnitude-squared coherence range between 0 and 1, in which values equal to one indicate high coherence and values equal to 0 no coherence.

We tested eight empirical relationships (here also referred to as models or statistical models) proposed in previous studies in the last 10 years for calculating (methane) ebullition fluxes (or in some models log-transformed fluxes) as a function of environmental variables (such as temperature, pressure, and others). Our aim was to explore if the ebullition variability at Passaúna reservoir can be described by the same set of variables as in other systems and to analyze to what extent these models can be applied across different systems to reproduce the temporal dynamics of ebullition. In this step we tested the models in two ways: with the same coefficients from the original studies and by recalculating the models’ coefficients based on our dataset for Passaúna reservoir. After that, we tested if the inclusion of additional environmental variables in different model set-ups can improve the models’ capability to reproduce the temporal dynamics of ebullition. Finally, we selected the model with the best performance in reproducing daily ebullition fluxes to test at which timescales the model could resolve ebullition variability.

For applying the empirical models proposed in the literature, we used the time series at the same time interval as reported in each respective study. The time intervals of estimated ebullition fluxes ranged between daily to biweekly. The models were tested to predict the accumulated fluxes (as a representation for mean/seasonal ebullition) and to reproduce ebullition temporal dynamics. The model’s capability to estimate the total accumulated methane flux was evaluated by calculating the relative error (Rel_error_) between measured and simulated values, in which a negative Rel_error_ indicates an overestimation by the model and a positive Rel_error_ an underestimation of accumulated fluxes. As continuous data are required for calculating accumulated fluxes, we restricted the calculation of Rel_error_ to two periods of continuous measurements: June 26^th^ 2018 –October 2^nd^ 2018 and December 14^th^ 2018 –February 05^th^ 2019. The model’s performance in reproducing ebullition temporal dynamics was evaluated through three metrics: coefficient of determination (R^2^), root-mean-square error (RMSE), and Nash-Sutcliffe efficiency (NSE) of measured and estimated non-log transformed fluxes. MatLab codes of the empirical models tested and models’ performance calculations are available in the Zenodo repository [47].

## Results

### Temporal dynamics of time-series

**Fig 2:**
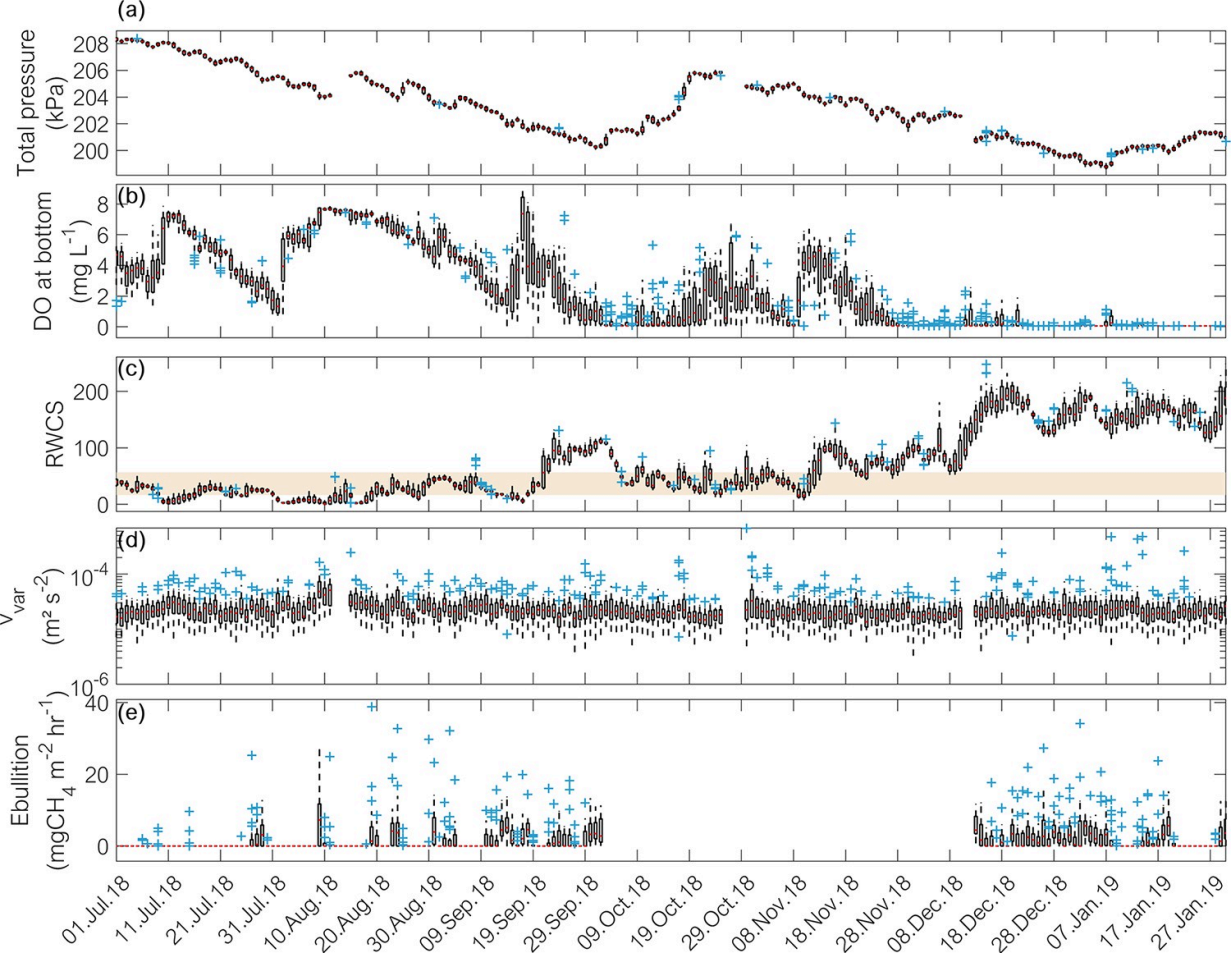
Box plots of daily variations in observed hourly time-series. (a) Total pressure (hydrostatic + atmospheric pressure). (b) Dissolved oxygen concentration measured near the bottom. (c) Relative Water Column Stability (RWCS, Eq 1). The shaded area represents values when the reservoir was partially stratified whereas higher values indicate stratified conditions. (d) Velocity variance near the bottom. (e) Methane ebullition flux at location P2. In all time series the blank spaces represent data gaps. The upper and lower limits of the boxes represent the 75^th^ and 25^th^ percentiles, respectively. The whiskers show the maximum and minimum values, the red line represents the median, and the blue crosses are outliers.

The measured variables were characterized by temporal variations of different magnitudes and at different frequencies (Fig 2). Pronounced sub-daily variability was observed in velocity variance, DO at the bottom, and in RWCS, whereas variations in total pressure occurred mostly over days. Seasonal and other longer-term trends were present in total pressure, DO and RWCS. Inter-dependencies between parameters were also observed, for instance the concentration of DO near the bottom was negatively correlated to RWCS (Spearman rank correlation rs = −0.76 with p-val < 0.05). In periods when the reservoir was mixed, the DO near the bottom tended to be higher, whereas the bottom water layer became anoxic during strong stratification (see Fig 2B after end of November 2018).

The ebullition fluxes at Passaúna reservoir were episodic and zero inflated. The fluxes were not normally distributed (Kolmogorov-Smirnov test), which can also be observed in the boxplots (see Fig 2E and S2 Fig), and thus, non-parametric methods were used for correlation analysis. The average ± standard deviation fluxes over the entire monitoring period at the monitoring locations were 24.2 ± 47.0 mgCH_4_ m ^– 2^ d ^– 1^ (n = 605, median 0, range 0 – 264.1 mgCH_4_ m ^– 2^ d ^– 1^) at site P1, 33.8 ± 49.3 mgCH_4_ m ^– 2^ d ^– 1^ (n = 680, median 3.8 mgCH_4_ m ^– 2^ d ^– 1^, range 0 – 238.7 mgCH_4_ m ^– 2^ d ^– 1^) at site P2, 18.9 ± 43.4 mgCH_4_ m ^– 2^ d ^– 1^ (n = 549, median 0, range 0 – 587.1 mgCH_4_ m ^– 2^ d ^– 1^) at the deepest site P3, and 19.1 ± 39.8 mgCH_4_ m ^– 2^ d ^– 1^ (n = 274, median 0, range 0 – 175.1 mgCH_4_ m ^– 2^ d ^– 1^) at site P4. Generally, ebullition was more frequent during summer and at the beginning of autumn (Dec–Apr), whereas during winter (Jun–Aug) the events of bubble release became less frequent, often resulting in daily median fluxes of zero during these months (see S2 Fig).

Spectral analysis was applied to time-series of methane ebullition at site P1, for which the longest continuous record (of almost one year from December 13^th^ 2018 to November 24^th^ 2019) was obtained. Most of the variance of the time-series was associated with high frequencies (> 10 ^– 4^ Hz, S3 Fig), i.e. at time scales shorter than 3 h. A minor peak of spectral variance was observed at frequencies corresponding to a 12 h period, however, from the wavelet transform we observed that the periodicity was not evenly distributed throughout the year, instead it was present mostly in the summer months from December to February, which were the months with more frequent ebullition fluxes.

**Fig 3:**
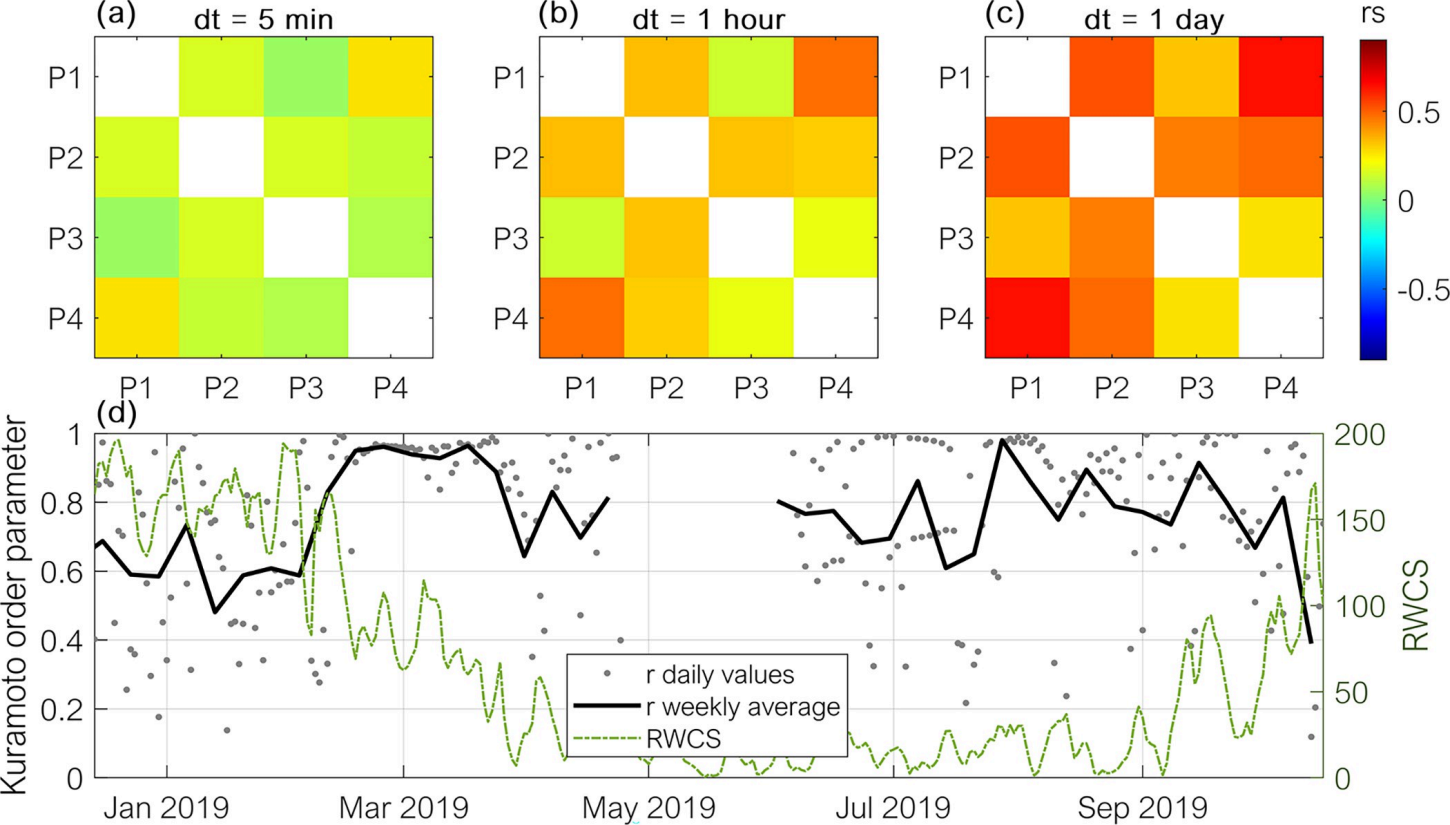
Correlation of ebullition fluxes among monitoring sites for different timescales and synchronization of fluxes. Correlation matrix of methane ebullition fluxes observed at the four monitoring sites (P1 –P4) at time intervals of 5 min (a), 1 hr (b), and 1 d (c) respectively. The colour indicates the Spearman rank correlation coefficient for a significance level of 5%. (d) Kuramoto order parameter (r, left-hand side axis) indicating the synchronization of daily ebullition fluxes at locations P1, P2, and P3 (grey dots) and weekly averaged r values (solid black line). Values of r = 1 indicate perfect synchrony whereas r = 0 indicates asynchrony of the fluxes. The green line shows the relative water column stability (RWCS, right-hand side axis).

The correlation of ebullition among locations was weak at the shortest time intervals (5 min, Spearman correlation rs < 0.3 and *p* < 0.05). Nevertheless, for increasing averaging intervals (1 hr and 1 d), the correlation between fluxes increased (Fig 3). The maximum correlation (rs = 0.65 p-val < 0.05) was observed between sites P1 and P4. For the periods with continuous and simultaneous measurements at locations P1, P2, and P3, the daily fluxes were highly synchronized with an average synchronization parameter r (Kuramoto order parameter) of 0.76 ± 0.23. The synchronization was highest from February to April 2019, whereas the lowest synchronization (mean r = 0.60) was in December 2018 (see Fig 3D).

The high degree of synchronization of the fluxes among locations suggests that ebullition was triggered by forcings acting over large spatial scales, which implies that the temporal dynamics of ebullition for time scales ≥ 1 d is similar among the monitoring locations. Therefore, for the statistical analysis of daily ebullition time-series in combination with environmental parameters described in the following sections, we only used the ebullition recorded at location P2, for which we had most valid data and for which most of the additional variables were measured in close vicinity (e.g., flow velocity, chl-a, water temperature, and DO).

### Ebullition drivers

Methane ebullition flux was significant positively correlated (Spearman correlation rs) with bottom temperature (rs = 0.35), as well as to RWCS (rs = 0.45) and to Schmidt stability (rs = 0.41). In addition, we found positive correlations between ebullition and velocity variance near the bottom (rs = 0.37) and with the mean current speed (rs = 0.29). Significant negative correlations of ebullition were observed with air pressure (rs = −0.49), water depth (rs = −0.24), dissolved oxygen concentration at the bottom (rs = −0.35), chlorophyll-a (rs = −0.29), and backscatter intensity in the bottom layer (rs = −0.23) (see S4 Fig for correlation matrix). In the principal component analysis (PCA) of ebullition with eleven variables (see S4 Fig) we observed two main groupings of the data points based on mixing conditions of the reservoir: mixed/partially stratified and stratified. Therefore, based on the PCA findings, the correlation analysis was additionally applied to subsets of the data, according to the prevailing stratification conditions (S4 Fig).

**Fig 4:**
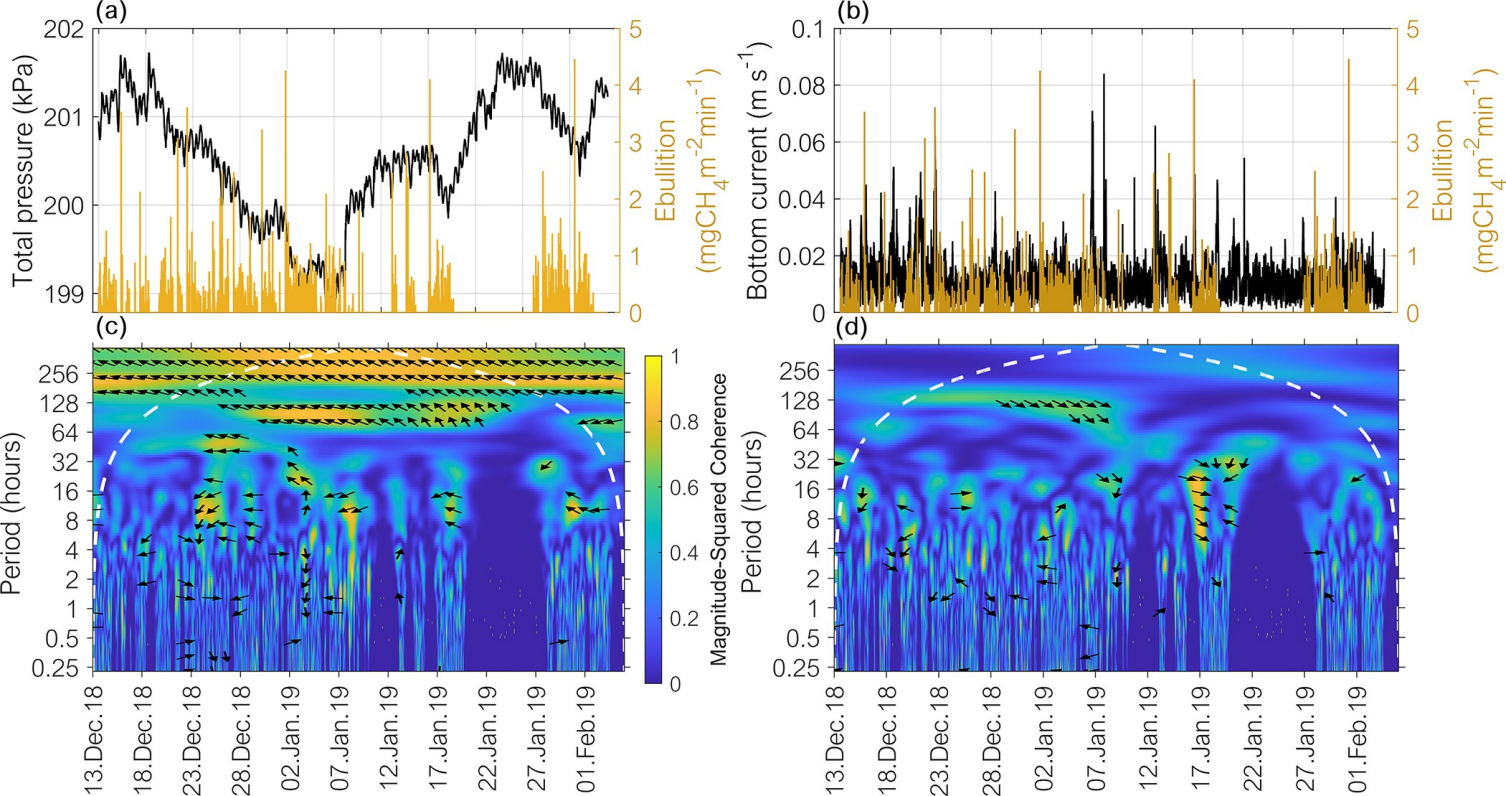
Time series of methane ebullition, total pressure, bottom current speed and results of the wavelet coherence analysis. (a) Total pressure (hydrostatic + atmospheric, left hand axis) at the sediment surface at location P2 and methane ebullition rates measured at location P2 (right-hand axis). (b) Near-bottom current speed (left-hand axis) and methane ebullition rates measured at location P2. (c) and (d) show the squared wavelet coherence magnitude of total pressure and ebullition, and bottom current speed and ebullition, respectively. In both panels, the dashed white lines mark the cones of influence, in which the areas above the lines represent unresolved timescales. The yellow colour represents regions of high coherence, while blue colour represents lower dependence between the time-series; the horizontal arrows pointing to the right and left directions (corresponding to 0 and π or 0 and ½ cycle) indicate that the variables (e.g. ebullition and driver) are in phase or in anti-phase respectively. Arrows pointing right-down or left-up indicate that ebullition occurs within a delay in relation to the driver (3/8 and 7/8 lag cycle, respectively). Whereas right-up (1/8 cycle) and left-down (5/8 cycle) indicates that ebullition is ahead of the driver. In all panels the time series are at 5 min time intervals.

When the reservoir was partially stratified, the dissipation rate of turbulent kinetic energy near the bottom was positively correlated with ebullition (rs = 0.31), whereas the bottom current velocity was positively correlated with ebullition when the reservoir was partially stratified (rs = 0.35) and stratified (rs = 0.30). Nevertheless, during the longest stratified period (December 2018 –February 2019), the wavelet coherence between time-series of methane ebullition and individual variables, such as total pressure and bottom current speed, varied with time and with the period, see in Fig 4. For the total pressure, there was high coherence with ebullition throughout the analysed time series for periods of approximately 8.5 d and 2.5 d, with the latter being more intensified between the end of December to the first week of January. Considering the bottom current, high coherence with ebullition was observed during short periods of less than 2.5 d. For both total pressure and bottom current the interrelation with ebullition occurred with a delay in respect to the variable (indicated by the left-up and right-down pointing arrows in Fig 4). For instance, considering the total pressure a delay ranging from 22.5 hrs to 76 hrs was observed (see Fig 4C, arrows indicating to approximate 3/8 to 1/2 cycle delay between ebullition and total pressure at the period of 60 to 204 hours). In summary, the strength to which ebullition correlated with these environmental controls depended on the considered timescale and on time of the year.

### Empirical models for ebullition prediction

**Fig 5:**
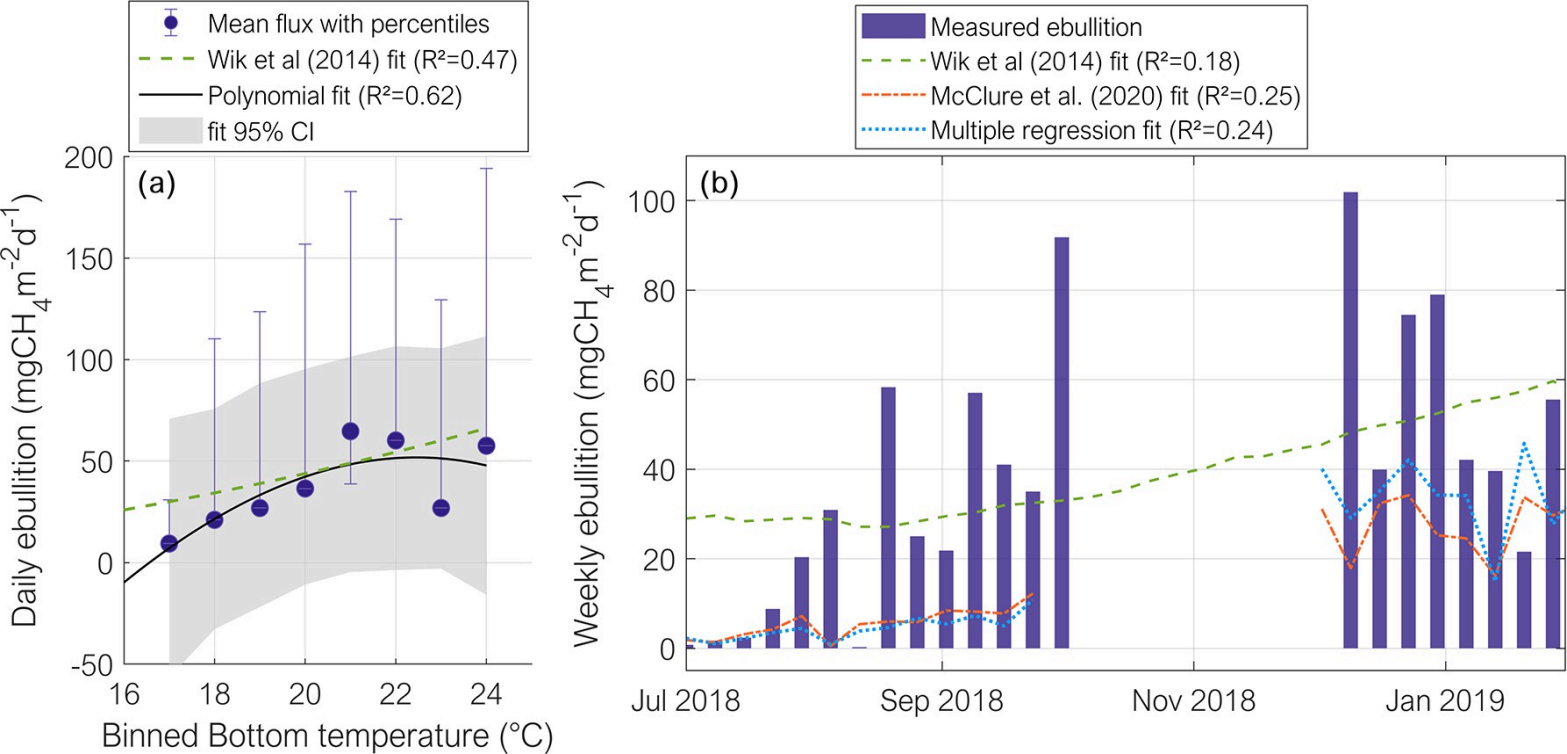
Empirical models from the literature tested for the prediction of ebullition. (a) Application of an empirical model (dashed green line) for predicting mean ebullition flux as a function of binned (by 1°C) sediment temperature (from [25]). The blue circles are the mean methane ebullition fluxes for each temperature bin and the error bars are the 10^th^ and 90^th^ percentiles; the solid black line is a polynomial fit to the data and the 95% confidence interval (CI) of the fit is shown by the grey shaded area. (b) Weekly-averaged time-series of methane ebullition flux (blue vertical bars) and simulated ebullition predicted by the empirical equations from [25] (dashed green line), from [29] (orange dot-dash line), and from a multiple regression with the same input variables as [29] (dotted blue line). The blank space between October and beginning of December 2018 is due to gaps in the measurements. The equations of all models are shown in S1 and S2 Tables.

Temperature (in the sediment or in water) and pressure (hydrostatic and atmospheric) were the most common ebullition predictors considered in empirical models presented in former studies (Table 1, and S1 Table). In general, the empirical models from previous studies had a poor performance in reproducing ebullition fluxes at Passaúna (R^2^ < 0.5 and Nash-Sutcliffe efficiency (NSE) < 0.3 between measured and simulated ebullition, Table 1). The best performance was achieved with the model that predicts mean ebullition fluxes solely as a function of binned sediment temperature (proposed by [25], which could explain 47% of mean methane ebullition variability). Nevertheless, the temporal dynamics of ebullition was not captured by the model, as sediment temperature was changing only slowly and averaging of fluxes based on binned temperature smoothes ebullition variability (Fig 5). An autoregressive model proposed by [29] for the simulation of reservoir-wide ebullition (average from different monitoring sites), which in addition to sediment temperature also considered wind speed, changes in atmospheric pressure, and ebullition from the previous time-step, could not well reproduce the flux variability of weekly-averaged fluxes (R^2^ = 0.25, Fig 5B). The same combination of variables with recalculated model coefficients did not improve the model capability to explain the variability of ebullition fluxes (R^2^ = 0.24, see S2 Table), indicating that more variables, or different models are required for describing ebullition variability in Passaúna Reservoir. All the other literature models tested had worse performance in predicting the temporal dynamics of ebullition. Considering the models with recalculated coefficients (see first part of S2 Table), the best performance (R^2^ = 0.32 and NSE = 0.31) was obtained for an Artificial Neural Network (ANN) model, in which daily time series of ebullition was explained as a function of the change in total pressure, total pressure and bottom temperature.

**Table 1:** Results of empirical models from the literature and the generalized additive models (GAM) implemented in this study for the prediction of ebullition fluxes at Passaúna Reservoir for different timescales. The timescales and predictor variables are provided. The model performance in reproducing the temporal dynamics of ebullition was evaluated using R^2^, RMSE, and NSE. The model performance for estimating the accumulated flux was evaluated using the Rel_error_. Extended tables are provided in the supporting material (S1 and S2 Tables) showing the equations of all models with additional information.

Reference	Timescale and variables	Performance for Passaúna
[25]	Temperature-binned daily ebullition	R^2^ = 0.47
		NSE = 0.23
	Predictors: binned sediment temperature	
		Rel_error_ = −24.3%
[30]	Daily methane ebullition time series	R^2^ = 0.03
	Predictors: change in total static pressure, total static pressure, and bottom temperature	NSE = −6.06
		Rel_error_ = 289.5%
[50]	Biweekly gas ebullition time-series	R^2^ = 0.20
		NSE = 0.09
	Predictors: sediment temperature	Rel_error_ = 39.4%
[50]	Biweekly methane ebullition time-series	R^2^ = 0.19
		NSE = 0.16
	Predictors: total phosphorous and sediment temperature	Rel_error_ = 30.1%
[18]	Temperature-binned daily ebullition	R^2^ = 0.39
	Predictors: methane ebullition at 20°C, site-specific temperature coefficient, and binned sediment temperature	NSE = 0.01
		Rel_error_ = −17.5%
[29]	Weekly methane ebullition time series	R^2^ = 0.25
	Predictors: methane ebullition from the previous time step, sediment temperature, wind speed, and change in atmospheric pressure	NSE = −0.11
		Rel_error_ = 62.5%
[28]	Daily methane ebullition time-series	R^2^ = 0.07
	Predictors: sediment temperature, sediment porosity, and organic matter content	NSE = 0.06
		Rel_error_ = 44.4%
[31]	Daily methane ebullition time series	R^2^ = 0.12
	Predictors: proportionality constant, pressure threshold, and total pressure	NSE = −0.005
		Rel_error_ = 9.8%
This Study	10-min methane ebullition time-series	R^2^ = 0.05
	Predictors: bottom current, dissipation rate near the bottom, sediment temperature, DO near bottom, atmospheric pressure, wind speed, RWCS, and total pressure.	NSE = 0.048
		Rel_error_ = −1.2%
This Study	Hourly methane ebullition time-series	R^2^ = 0.19
	Predictors: bottom current, velocity variance near the bottom, dissipation rate near the bottom, sediment temperature, DO near bottom, atmospheric pressure, wind speed, RWCS, and total pressure	NSE = 0.19
		Rel_error_ = −1.4%
This Study	Daily methane ebullition time-series	R^2^ = 0.70
		NSE = 0.69
	Predictors: bottom current, velocity variance near the bottom, dissipation rate near the bottom, sediment temperature, DO near bottom, atmospheric pressure, wind speed, RWCS, and total pressure	Rel_error_ = −0.28%
This Study	Weekly methane ebullition time-series	R^2^ = 0.96
	Predictors: bottom current, velocity variance near the bottom, dissipation rate near the bottom, sediment temperature, DO near bottom, atmospheric pressure, wind speed, RWCS, and total pressure	NSE = 0.96
		Rel_error_ = 0.01%

**Fig 6:**
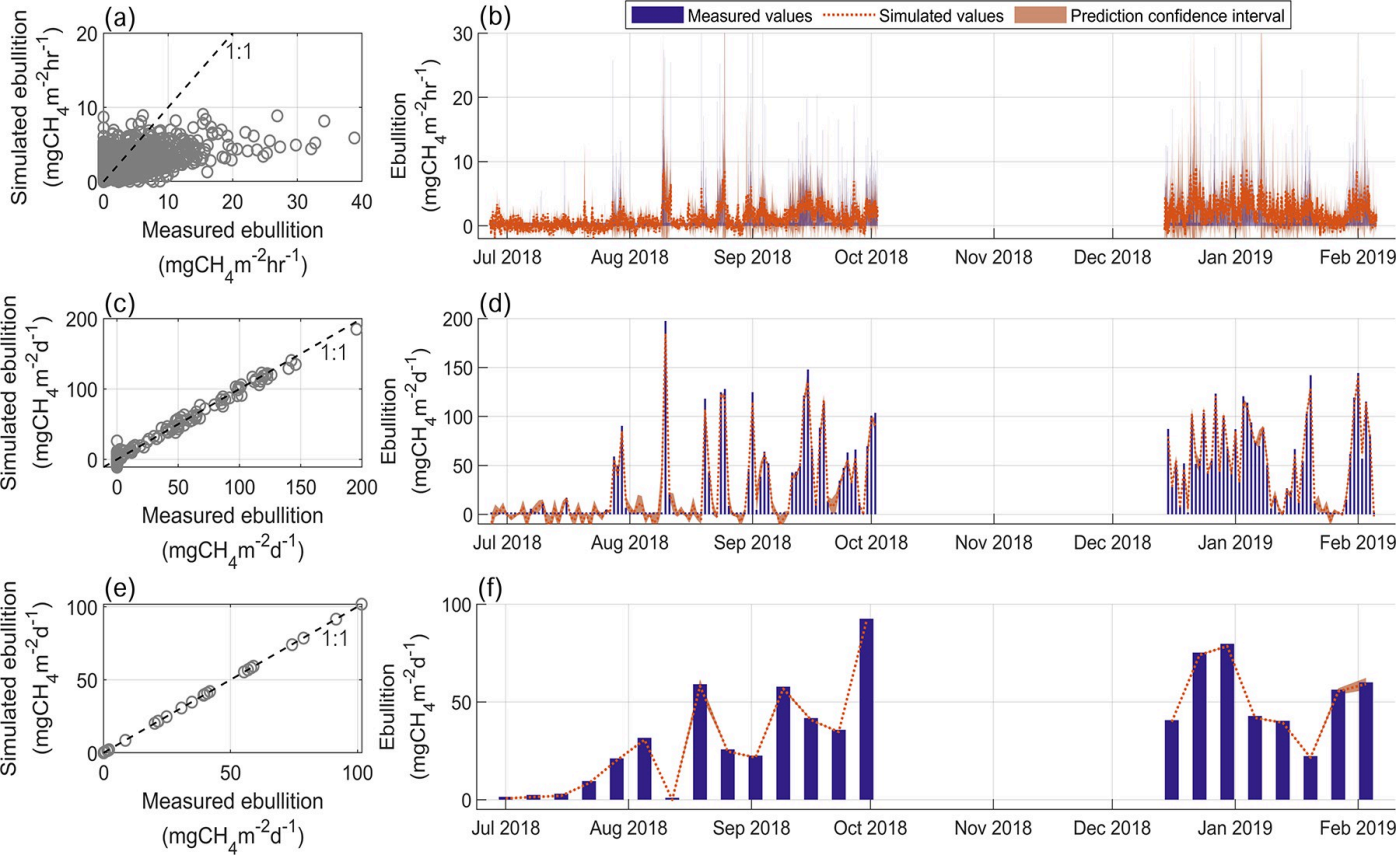
Results of a generalized additive model (GAM) for reproducing methane ebullition at location P2 for hourly (a and b), daily (c and d), and weekly (e and f) time scales. The mean ebullition was predicted as a function of bottom current, velocity variance and energy dissipation rates near the bottom, sediment temperature, DO near the bottom, atmospheric pressure, wind speed, relative water column stability (RWCS), and total pressure. Model’s equations and performance statistics are presented in S2 Table in the supporting material. Panels a, c, and e show scatter plots of simulated versus measured ebullition rates. Panels b, d, and f show time-series at the respective time steps (1 hr, 1 d, 1 week) of measured and simulated ebullition rates (as well as the prediction 95% confidence intervals, see legend).

The performance of new empirical models for the prediction of daily time-series of ebullition tested with various combinations of input variables, varied from a R^2^ of 0.10 to 0.70 (see S2 Table for all the new models implemented). The best result was obtained from a generalized additive model (GAM, see Fig 6 and Table 1), in which methane ebullition was explained using a sum of univariate shape functions of predictors. The input predictors were bottom current, velocity variance at the bottom, energy dissipation rate near the bottom, sediment temperature, DO at the bottom, atmospheric pressure, wind speed, relative water column stability (RWCS) and total pressure. The same set of variables in a similar model could explain 96% (and NSE = 0.96) of the variability of weekly ebullition time series. Nevertheless, as we increased the temporal resolution of the time series (time steps of 1 hr and 10 min), the models could not reproduce most of the variability (R^2^ = 0.19 NSE = 0.19 and R^2^ = 0.05 NSE = 0.048 respectively). The predicted R-squared values were additionally calculated for the GAM models as an indicator of model overfitting. The values obtained for the predicted R-squared were below zero, which can be interpreted as equal to zero, indicating potential overfitting of the models (see S2 Table).

Considering the models for estimating the total accumulated methane flux (S1 and S2 Tables), the models tested from the literature could be improved by recalculating the model coefficients for data from Passaúna Reservoir. For instance the polynomial fit proposed by [25] had a Rel_error_ of – 24.3% in reproducing accumulated ebullition, whereas with the recalculated polynomial fit we obtained a Rel_error_ of 2.06x10 ^– 13^% (see Fig 5A). Similarly, the artificial neural network originally trained in [30] resulted in an overestimation of 289.5% (Rel_error_) of accumulated fluxes at our study site, however, with a retrained neural network using the same input variables the relative error was reduced to 4.4%. Rel_error_ could be further reduced in the new models developed for the Passaúna dataset, with the lowest error obtained also from the generalized additive model (Rel_error_ = −0.28%, for daily time-series). For shorter time scales, the Rel_error_ of the generalized additive model increased to −1.4% and −1.2% for the hourly and 10 min resolution (Table 1).

## Discussion

### Ebullition temporal patterns and drivers

Ebullition flux in freshwater reservoirs is considered as being stochastic in nature [4, 51, 52], which turns both measurements and prediction of emissions challenging. At Passaúna Reservoir, ebullition fluxes were highly variable at different time-scales (minutes to months) with most of the temporal variance occurring at sub-daily timescales, as similarly reported for a river impoundment in Germany [16]. As a result of the episodic occurrence of ebullition, the time series were zero-inflated leading to frequent zero median fluxes throughout the monitoring period (from February 2017 to February 2020). On the one hand, median values are preferred over mean values for non-normally distributed data, such as ebullition [2]. On the other hand, median values lead to zero emissions estimates when using high temporal resolution measurements (time-scale ≤ 1 d). These results confirm that the use of cumulative fluxes is more appropriate for representing ebullition [53], and therefore, the importance of continuous measurements.

Considering seasonal variations, the extended time series of ebullition fluxes analyzed in the present study, confirm previous results [41], showing that ebullition at Passaúna is intensified during periods when the reservoir is stratified (also confirmed by the PCA analysis, S4 Fig). This can be explained by a combination of higher sediment temperature (up to 23°C), reduced methane loss from the sediment due to diffusive transport [54], and reduced methane oxidation as the water overlaying the sediment has lower dissolved oxygen concentrations (< 2 mg L^−1^) during stratification; all aspects favouring methane production and accumulation in the sediment.

The stochastic nature of ebullition fluxes is caused by the multitude of processes affecting the temporal and spatial dynamics of gas production, accumulation, and release from the sediment. In addition to stratification, other variables that have been reported for other water bodies were significant drivers of ebullition at Passaúna Reservoir, such as bottom water temperature [18, 50], atmospheric and hydrostatic pressure [14–16], and potential methane production [55]. Nevertheless, we would like to highlight interesting aspects found at Passaúna reservoir.

Surprisingly, ebullition was negatively correlated with chlorophyll-a concentrations (rs = −0.29). The presence of chlorophyll-a indicates fresh labile organic matter, which can enhance methanogenesis and sediment methane production [56], when deposited on the sediment. Other studies have found a positive relationship between ebullition and chlorophyll-a concentrations across different aquatic systems [3, 50]. At Passaúna reservoir, chlorophyll-a concentrations were reported to be higher after periods of mixing during autumn and winter [37], when sediment temperature was lower and the bottom water was oxic (see S5 Fig). These conditions might lead to reduced methane production and enhanced methane oxidation. In addition, the diffusive transport of methane across the sediment water interface is enhanced during mixing conditions, resulting in lower ebullition fluxes [22]. In this way, the positive effect of higher chlorophyll-a could have been off-set by the combination of variables leading to a flux reduction. Two additional aspects are also important to be noted. First, correlations were calculated considering daily time-series, however the effects might be observed only for longer time intervals (months or seasons). Secondly, the chlorophyll measurements were done at 1.5 m below the water surface at site P2, and most of the chlorophyll deposition on the bed is expected to occur in the region around location P3 [37], nevertheless we also obtained significant negative correlation (rs = −0.15) between chlorophyll-a and ebullition flux measured at site P3.

Our extended set of variables facilitated analyses of ebullition fluxes in relation to the hydrodynamic conditions in the reservoir. Although stronger stratification (higher S_T_ and RWCS) occurred during the warmer summer months, the mixing periods at Passaúna reservoir were not restricted to one specific season, as the reservoir is classified as warm-polymictic [39]. Furthermore, according to [39], higher flow velocities associated with wind-driven currents were more frequent during stratified periods, whereas dissipation rates near the bottom were higher during mixed conditions. Consequently, we found that the effect of bottom currents and dissipation rates on ebullition were linked to the stratification conditions. During periods when the reservoir was stratified and partially stratified, the bottom currents were positively correlated to ebullition flux (rs = 0.36 and p < 0.05, S4 Fig), in which ebullition events were occurring shortly after increased bottom currents (Fig 4B and 4D). A previous study showed that bottom currents can trigger bubble release, if the gas reservoir in the sediment is not depleted [17]. When the amount of gas accumulated in the sediment was reduced, such as during mixing, bottom currents cannot trigger ebullition. We used the energy dissipation rates near the bottom as an indicator for the intensity of turbulence near the sediment [57], and we expect a trade-off between two contrasting effects of turbulence on ebullition. On one hand, mechanical disturbances at the sediment surface can cause bubble release, whereas on the other hand, the increase in diffusive transport by near-bed turbulence can cause an increased loss of methane from the sediment by diffusive fluxes [22]. In fact, significant correlation (rs = 0.31) between ebullition and dissipation rates was only detected when the reservoir was partially stratified, mainly between July-August of 2018 (see details in S6 Fig). During this period, the higher dissipation rates triggered ebullition, whereas during stratification, when the dissipation rates were generally lower, the negative sign of correlation suggests a dampening effect on ebullition.

Lastly, we found that the strength and direction of the effects of environmental variables on ebullition changed over time. Former studies proposed that the temporal dynamics of ebullition in different systems could be classified as controlled by forcing (ebullition triggers), or controlled by methane production in the sediment [16]. In systems with continuously high methane production and high-frequency forcing, ebullition fluxes are continuously high. In systems with lower production and low frequency forcing, in contrast, ebullition dynamics is mainly controlled by the dynamics of the forcing. We argue that Passaúna Reservoir is switching between both states depending on stratification conditions. As the variation of sediment temperature at Passauna was relatively small (16–23°C), temperature might not be the main control on methane production, but as discussed above, the loss of methane from the sediment (e.g. increased oxidation and diffusion) are expected to be the largest during the moments of reduced ebullition. Thus, during the periods with reduced methane accumulation in the sediment (e.g. during mixed conditions), ebullition dynamics is governed by the forcing, in which the high synchrony of ebullition among locations during this period suggests that large-scale forcings are dominant. However, during conditions favouring methane production and accumulation (e.g. with reduced losses other than ebullition), the site-specific characteristics (such as water depth and sediment properties) become also relevant in modulating ebullition dynamics, and thus reducing the synchrony of ebullition events among locations (see Fig 2, we obtained significant negative correlation rs = −0.37 between weekly values of RWCS and the Kuramoto order parameter characterizing the spatial synchrony of ebullition fluxes).

### Empirical models for representing mean fluxes

The seasonal variations of mean ebullition fluxes could be well predicted by a simple empirical model considering binned sediment temperature proposed by [25] (R^2^ = 0.47 and Rel_error_ = −24.3% for the original polynomial fit and R^2^ = 0.62 and Rel_error_ = 2.06x10 ^– 13^% for the refitted model). The good fit of the model to our dataset from a subtropical man-made reservoir was surprising, as the model was originally obtained for data from subarctic postglacial lakes with contrasting characteristics. The model’s capability of predicting ebullition across different systems, would point to the possibility of a universal model. Nevertheless, [18] showed that a single model for predicting the varying magnitudes of ebullition across different systems as a function of temperature is not possible. However, the exponential dependence of ebullition on temperature is shared among the different systems [18], which was also observed at Passaúna Reservoir. The mean fluxes for binned sediment temperature were minimum for temperatures lower than 18°C (Fig 5A). Using the modified Arrhenius equation as proposed by [18], we could explain 39% of mean ebullition as a function of binned temperature, however with an overestimation of accumulated fluxes by – 17.5% (Table 1).

The difficulty in transferring models across systems was also observed for the prediction of accumulated fluxes. On overall, the models from previous studies failed in predicting the accumulated fluxes (Rel_error_ from −346.9% to 289.5%, S1 Table). The model proposed by [31] based on total pressure could estimate the accumulated fluxes with an underestimation of 10%, however the model parameters were site specific (to Passaúna), which explain the improved model performance (Table 1). Nonetheless, the range of relative errors obtained from the prediction of accumulated fluxes were lower (from −2.8% to 48.1%) for the new models implemented, which can result from the combination of the addition of new ebullition predictors, the model’s capability of capturing the short-term temporal dynamics of ebullition (and thus, reducing accumulated errors), and the tuning of model parameters to our data.

### Empirical models for representing the temporal dynamics of ebullition

The characteristics of ebullition time-series of episodic/pulse events and being zero-inflated, in combination with the complex connection to various drivers, pose difficulties in reproducing the temporal dynamics of ebullition fluxes and its magnitude using empirical approaches. Nevertheless, previous studies could successfully reproduce the temporal dynamics of ebullition by considering only atmospheric pressure (R^2^ = 0.87) [31, 58], or a combination of more variables. For instance, [29] reproduced ebullition time series with an autoregressive model using sediment temperature, wind speed, change in atmospheric pressure with good agreement (R^2^ = 0.86). Eight models, proposed for other aquatic systems including lakes and reservoirs, were directly applied to Passaúna reservoir. The objective was to assess the extent to which these models can be employed across different systems to simulate ebullition fluxes, and to determine if they share similar controls on the fluxes. When applied to Passaúna reservoir, the empirical models from other systems resulted in a poor representation of ebullition variability, with the best results for reproducing ebullition temporal dynamics obtained by the equation proposed by [29] (R^2^ = 0.25 and NSE = −0.03 in Table 1 and S1 Table).

Although the simulated fluxes were still within the range of the measured values (Fig 4B), the results indicated a weak transferability of empirical models from one system to another. In addition, other factors that may influence ebullition fluxes, such as mixing regimes, watershed area and characteristics, or the number of sampled sites, are commonly not considered in the different models. In a second step, the models proposed from the literature were refitted to our dataset. However, by readjusting the models’ coefficients to the observations at Passaúna, the combination of the same variables could represent only a fraction of daily methane ebullition variability (R^2^ < 0.35, see S2 Table). Therefore, other factors are playing an important role in controlling ebullition dynamics in Passaúna Reservoir.

In a third step, new empirical models were implemented and tested for prediction of ebullition by including additional variables. A combination of nine input variables (including temperature, pressure, DO, velocity, and turbulence) in a generalized additive model could well capture the main temporal dynamics in daily ebullition time-series (R^2^ = 0.70 and NSE = 0.69) and in weekly time-series (R^2^ = 0.96 and NSE = 0.96), however, with poor performance for reproducing hourly time-series (R^2^ = 0.19 and NSE = 0.19, Fig 6). The difficulty in reproducing ebullition with high temporal resolution is in part caused by the fact that with short time intervals the episodic behaviour of the time series is mostly pronounced, and also, because time mismatches when synchronizing several time-series measured by various devices are more likely to affect the results [13].

According to [32], the transferability of empirical model for prediction of ebullition dynamics is likely to be weak even in the same system, whereas a continuous update of model constants by the inclusion of new measurements can improve the model performance in predicting ebullition. In this direction, we showed that ebullition was correlated with several parameters, however the strength of the correlation with each parameter was changing over time and was controlled by stratification conditions. For some variables, such as for the dissipation rate of turbulent kinetic energy, even the direction of the correlation was time dependent. Therefore, linear models will most likely fail in reproducing ebullition temporal patterns with a good performance, which can also result in a poor estimation of the accumulated fluxes and mean ebullition rates.

### Broader Implications, limitations, and further studies

Statistical analysis is a key approach for addressing highly variable processes, such as ebullition. Similar to rainfall, ebullition is characterized by episodic events of varying intensity, duration, and frequency of occurrence [13, 41]. However, there is still no established and standard approach to measure and describe ebullition statistics in terms of long term and continuous measurements, timescales of analysis, and representation of fluxes. Time series analysis can provide insights into the underlying processes and controls on methane ebullition in freshwater reservoirs and therewith improve the understanding of its temporal dynamics. Further analysis can bring additional insights into ebullition timescales, for instance by exploring the fractal dimensions in ebullition time series, similarly to analyses of rainfall data [59]. When applied to time series, fractal dimensions allow to connect information and to identify repeating complexities and patterns across different timescales [60], which in turn, might be useful for identifying the relevant time scales of ebullition across different systems.

Here we evaluated the temporal dynamics of ebullition mostly based on measurements from a single monitoring site (P2). We argued that it is representative for the other three monitoring sites in the Reservoir, as we found high synchronization of ebullition fluxes across the sites at daily timescales. However, it is important to mention that at shorter timescales (< 1 d), the degree of synchronization and the correlation of fluxes among the monitoring sites decreased. In addition, as found by [29] for a shallow reservoir (maximum depth of 9.3 m), the relevance of ebullition predictors differed between different regions of the reservoir, mainly longitudinally. Therefore, we speculate that for shorter timescales (< 1 d), shallow regions of the reservoir (upstream of site P1), and during periods when the fluxes among locations were asynchronous, the temporal variability of ebullition varied among locations, which could not be resolved in the available data.

The combination of different conditions (e.g., elevated methane production, higher temperature, reduction in water pressure) can culminate to hot moments and hot spots of methane emissions [3, 4]. Being able to predict the timing of such events is a basic initial step towards the development and implementation of methods for handling methane in water bodies. For instance, as summarized in [35], several of methane mitigation strategies rely on capturing and treating the methane, or are applicable only when methane gas fraction exceeds 1% (this methane fraction is easily found in rising bubbles). Empirical relationships can be fitted to capture and reproduce the temporal dynamics of ebullition as a function of a set of known variables and be applied to identify the hot moments of methane venting from aquatic systems. The advantages of data-driven models in comparison to mechanistic approaches, are that data-driven models generally require less computational power and are more flexible in terms of required input parameters, whereas process-based models might resolve complex physical and biogeochemical processes of methane formation and bubbles dynamics to simulate ebullition, which requires specific measurements for model calibration and validation [20, 24, 61]. In addition, statistical approaches and data-driven models are widely applied for filling measurement gaps in different fields, which can also be used for the case of ebullition for complementing existing measurements that are sparse in time.

We found that existing empirical models cannot be transferred across different systems. Similar to mechanistic approaches, model parameters need to be tuned to each system. The capability of empirical models for reproducing ebullition temporal dynamics was linked to the timescale under consideration. The models tested in this study had increasing difficulty in reproducing high temporal (< 1 d) variabilities of ebullition, since the randomness of the processes and uncertainties in the measurements increase with increasing temporal resolution. This is an important limitation because most of the ebullition variance was found at sub-daily timescales. In this regard, a mechanistic approach coupled with empirical models can potentially improve the predictions, which would have to be tested in future studies. Lastly, while a model based only on sediment temperature could estimated the total accumulated ebullition with a small relative error (< 1%), a more complex model was necessary to capture the temporal dynamics of ebullition. Considering that ebullition does not respond linearly to its forcing, models that are capable of handling non-linearities among variables are better candidates to capture and to reproduce ebullition flux dynamics. Nonetheless, it is important to note that in the case of the tested generalized additive models (GAM, see S2 Table), the results indicated potential overfitting of the models to the data. Model overfitting is an important aspect, as it affects model’s ability to generalize, consequently impacting its transferability. Therefore, model overfitting should be further investigated. For the case of GAM models, a penalty function can be used to the likelihood estimation to avoid overfitting [62].

Furthermore, we identified that the strength of ebullition drivers was modulated by stratification conditions in the reservoir. Therefore, the implementation of different empirical models depending on the mixing conditions can potentially improve the prediction of ebullition fluxes, which can be investigated in futures studies.

Lastly, the wavelet coherence analysis between ebullition and total pressure, as well as bottom current (Fig 4), revealed an existing delay of ebullition events relative to the drivers. Given the irregularities in these delays over time and their varying durations (as illustrated in Fig 4), they were not accounted for in the empirical models tested in this study. Therefore, its potential inclusion in empirical models remains an open issue to be further investigated, which may provide valuable insights into refining and enhancing the predictive capabilities of the models.

## Conclusion

We analysed high frequency ebullition measurements over three years of monitoring to understand the influence of various controls on its temporal dynamics and to evaluate the application of empirical models for reproducing the dynamics of ebullition fluxes across different aquatic systems and timescales. Vertical thermal stratification was an important modulator of the temporal dynamics of ebullition, impacting the strength and in some cases also the direction of the correlation between forcing and fluxes. The capability of reproducing and predicting ebullition fluxes from water bodies is relevant both in terms of estimating greenhouse gas budgets and for implementing mitigation strategies. Although empirical models are useful in understanding the drivers governing ebullition in aquatic system, to fill measurements gaps, and as a supporting tool for reservoirs management, they had a weak transferability from one system to another, which is caused by the complex interactions between ebullition and its controls. Weekly to daily temporal variability of ebullition could be well explained (R^2^ = 0.96 and R = 0.70) by a site-specific, generalized additive model considering nine input variables (bottom current, velocity variance at the bottom, dissipation rate near the bottom, sediment temperature, DO concentrations near the bottom, atmospheric pressure, wind speed, relative water column stability (RWCS) and total pressure). On longer time scales, the total accumulated ebullition flux could be well estimated by the generalized additive model (Rel_error_ = −0.28%). The relative error is further reduced (Rel_error_ = 2.06x10 ^– 13^%) for a model based on sediment temperature only, however at the expense of not resolving temporal variability. Our results demonstrate that there is certainly no unique solution for predicting methane ebullition dynamics in aquatic systems, however, we suggest further studies to explore simplified approaches for reproducing ebullition temporal dynamics (such as exploring the fractal dimensions of ebullition), and to combine empirical models with mechanistic-based approaches.

## Supporting information

S1 Fig Overview of measurements conducted at Passaúna reservoir during three years of monitoring. The Acoustic Doppler Current Profiler (ADCP) recorded velocity profiles, acoustic backscatter, and water depth; the dissolved oxygen loggers (near reservoir bed and near surface) recorded dissolved oxygen concentrations and water temperature; a chain of thermistors recorded the vertical distribution of water temperature; chlorophyll-a concentrations were recorded by a Fluorometer; the weather stations recorded wind velocity and direction, solar radiation, air temperature, atmospheric pressure, and humidity; and the Automated Bubble Traps (ABTs) recorded gas ebullition. The sensor locations are shown in Fig 1. The breaks within the horizontal lines indicate data gaps.
(TIF)

S2 Fig Boxplots of monthly methane ebullition fluxes obtained from daily estimates at the four sampling locations between January 2017 and March 2020. (a) site P1 (Park); (b) site P2 (Intake); (c) site P3 (Dam); (d) site P4 (side arm). In all time series the blank spaces are data gaps. The upper and lower limits of the boxes represent the 75^th^ and 25^th^ percentiles, respectively. The whiskers show the maximum and minimum values, the red line represents the median, and the blue crosses are outliers.
(TIF)

S3 Fig Variance-preserving power spectra and wavelet transform of methane ebullition time-series at site P1. (a) Variance-preserving power spectra of methane ebullition flux at site P1 with a sampling period of 5 min. (b) Wavelet transform of methane ebullition time-series at site P1. The colour represents the absolute value of the continuous wavelet transform in which the yellow colour indicates regions of periodic components and blue regions of low periodic components. The dashed white line shows the cone of influence, in which the areas below the line represent unresolved time scales. For both graphs the period of data used is from December 13^th^ of 2018 to November 24^th^ of 2019.
(TIF)

S4 Fig Correlation matrix and principal component analysis of ebullition and environmental variables time-series at location P2. (a) Correlation matrix (Spearman rank correlation) of daily methane ebullition fluxes at location P2 and daily averages of measured environmental parameters. The colour scales with the magnitude of the correlation coefficient (see legend) for a significance level of 0.05 (white boxes refer to no significance correlations with p > 0.05). The first column is the correlation with the complete data set whereas for the 3 other columns the data set was divided according to the prevailing stratification conditions. PSWI refers to the potential methane flux at sediment water interface; RMSV is the Root Mean Square Velocity; W temperature (bottom or surface) is water temperature (near reservoir bed or below the water surface); and Chla is Chlorophyll-a concentration. Total pressure is the sum of hydrostatic and atmospheric pressure. (b) Results of a Principal Component Analysis (PCA) of selected variables with the first two principal components. The dots are data points (daily values) which were grouped based on the stratification conditions by different colours (see legend). Components 1 and 2 explained 47.8% and 15.5% respectively of the data set variability.
(TIF)

S5 Fig Scatter plots of ebullition, sediment temperature, and chlorophyll-a concentration. (a) Scatter plot of chlorophyll-a and bottom temperature with the colour scale representing the difference of dissolved oxygen concentrations measured near the surface and near the bottom of the reservoir (see legend). (b) Scatter plot of chlorophyll-a and bottom temperature with the colour scale representing the methane ebullition flux (see legend). (c) Mean methane ebullition flux versus binned chlorophyll-a (by 1 μg L^-1^). The blue circles show the mean flux for each Chla bin and the error bars are the 10^th^ and 90^th^ percentiles; the solid black line is an exponential fit (*y* = 145.7 *e*^−0.24 *x*^) to the data and the 95% confidence interval (CI) of the fit is shown by the grey shaded area. All plots are based on daily mean values at monitoring site P2.
(TIF)

S6 Fig Scatter plot and wavelet coherence analysis between total dissipation rates and ebullition. (a) Scatter plot between daily methane ebullition and energy dissipation rates at location P2 grouped by the stratification classification (see colours and legend). Spearman correlation coefficients and significance levels between ebullition and log_10_-transformed dissipation rates are also shown in the legend. (b) and (c) shows the wavelet coherence analysis between total dissipation rates and ebullition for two intervals of measurements: mixed/partially stratified in (b) and stratified in (c). In both figures, the dashed white lines are the cones of influence, in which the areas above the lines represent time and scales with no dependence in the series. The yellow colour represents regions with high coherence, while blue colour represents lower dependence between the time-series. For both panels the time series are at 10 min time intervals (as the highest time resolution available of dissipation rates are at 10 min time steps).
(TIF)

S1 Table Summary of empirical models from other studies tested for the prediction of ebullition fluxes (y in mL m^-2^ d^-1^ or methane flux in mg CH_4_ m^-2^ d^-1^) at Passaúna reservoir. The timescale of each model is provided in the column ‘Variables’. Additional information about the application of the models is provided as footnotes below the table. The model performance on predicting ebullition was evaluated considering the coefficient of determination (R^2^) of a linear fit between measured and simulated ebullition, the root-mean-square error (RMSE), and the Nash-Sutcliffe efficiency (NSE). The relative error (Rel_error_ in blue color) was calculated between measured and simulated total accumulated flux, in which negative values indicate an overestimation by the model and positive values an underestimation.
(PDF)

S2 Table Empirical models tested for the prediction of ebullition flux (y in mL m^-2^d^-1^ or methane flux in mg CH_4_ m^-2^ d^-1^) based on data from Passaúna reservoir. The timescale of each model is provided in the column ‘Variables’. Additional information about the application of the models are provided as footnotes below the table. The model performance in predicting ebullition was evaluated considering the coefficient of determination (R^2^) of a linear fit between measured and simulated ebullition, the root-mean-square error (RMSE), and the Nash-Sutcliffe efficiency (NSE). The relative error (Rel_error_) was calculated between measured and simulated total accumulated flux, in which negative values indicate an overestimation by the model and positive values an underestimation.
(PDF)
