# Supplementary figures and images for: Exploring the temporal dynamics of methane ebullition in a subtropical freshwater reservoir
Source: PLoS One. 2024 Mar 27;19(3):e0298186. doi: 10.1371/journal.pone.0298186 (PMC10971506; doi:10.1371/journal.pone.0298186)

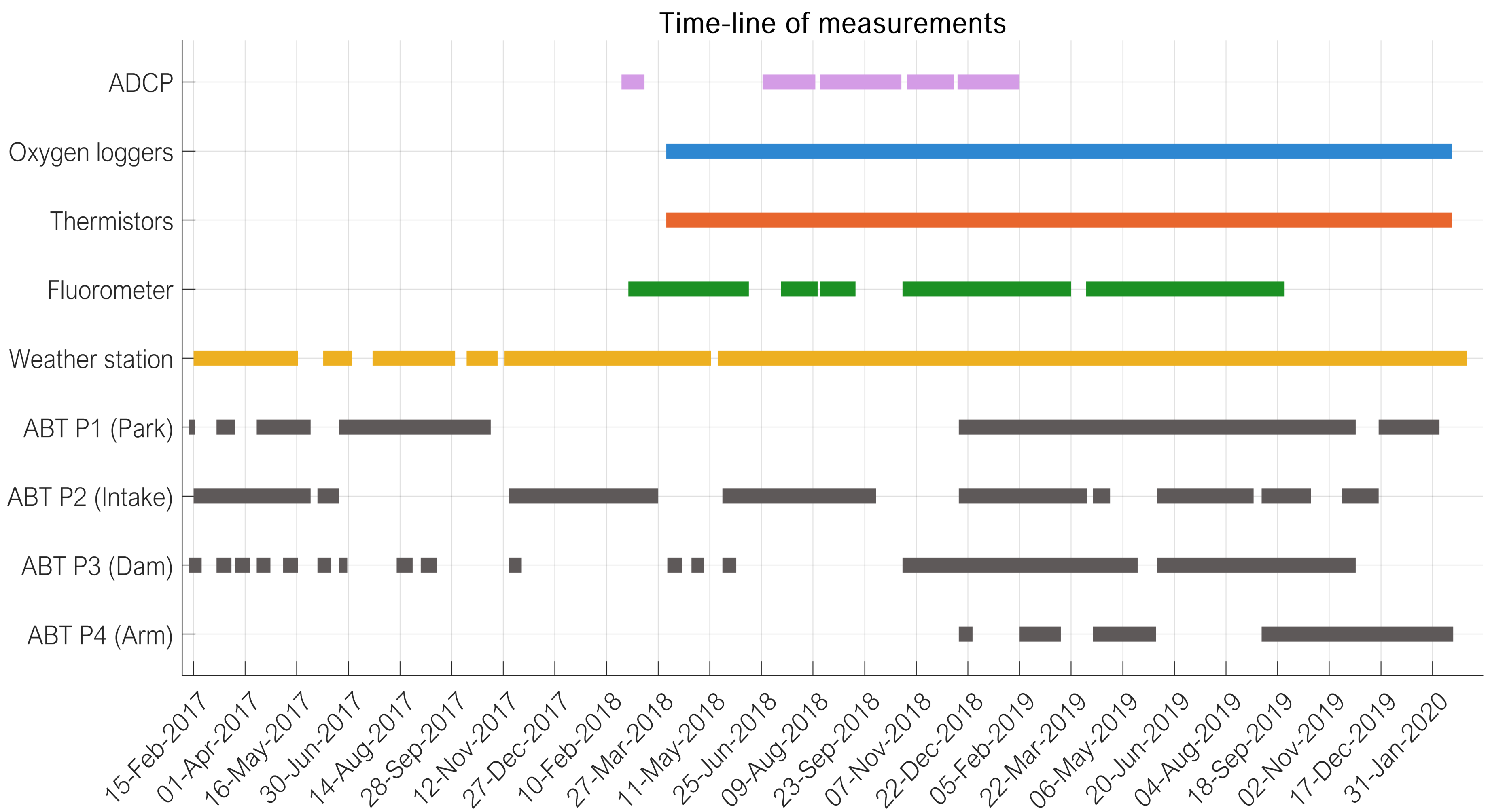

Supplement: S1 Fig — The Acoustic Doppler Current Profiler (ADCP) recorded velocity profiles, acoustic backscatter, and water depth; the dissolved oxygen loggers (near reservoir bed and near surface) recorded dissolved oxygen concentrations and water temperature; a chain of thermistors recorded the vertical distribution of water temperature; chlorophyll-a concentrations were recorded by a Fluorometer; the weather stations recorded wind velocity and direction, solar radiation, air temperature, atmospheric pressure, and humidity; and the Automated Bubble Traps (ABTs) recorded gas ebullition. The sensor locations are shown in Fig 1. The breaks within the horizontal lines indicate data gaps. (TIF) [file pone.0298186.s001.tif]

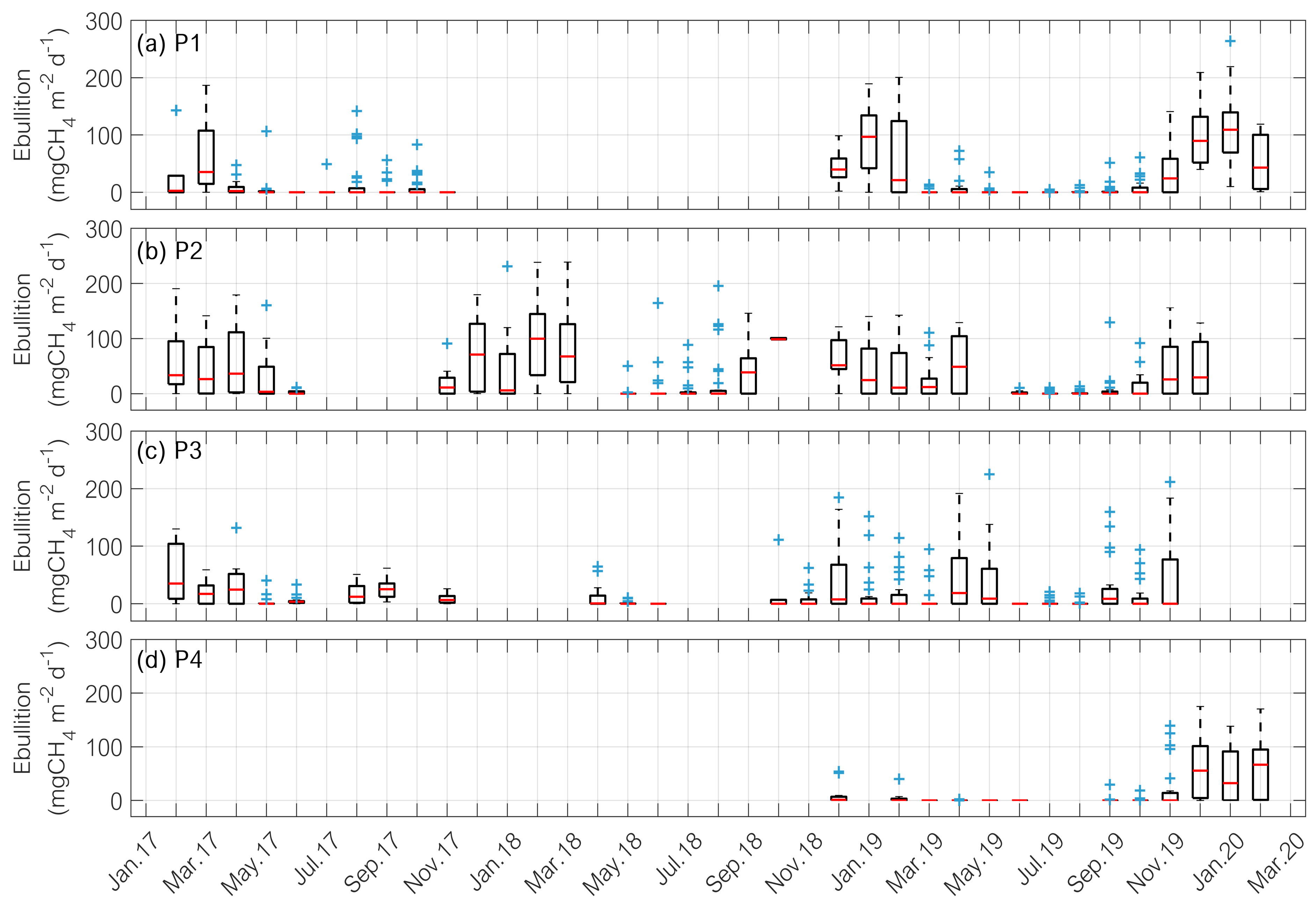

Supplement: S2 Fig — (a) site P1 (Park); (b) site P2 (Intake); (c) site P3 (Dam); (d) site P4 (side arm). In all time series the blank spaces are data gaps. The upper and lower limits of the boxes represent the 75th and 25th percentiles, respectively. The whiskers show the maximum and minimum values, the red line represents the median, and the blue crosses are outliers. (TIF) [file pone.0298186.s002.tif]

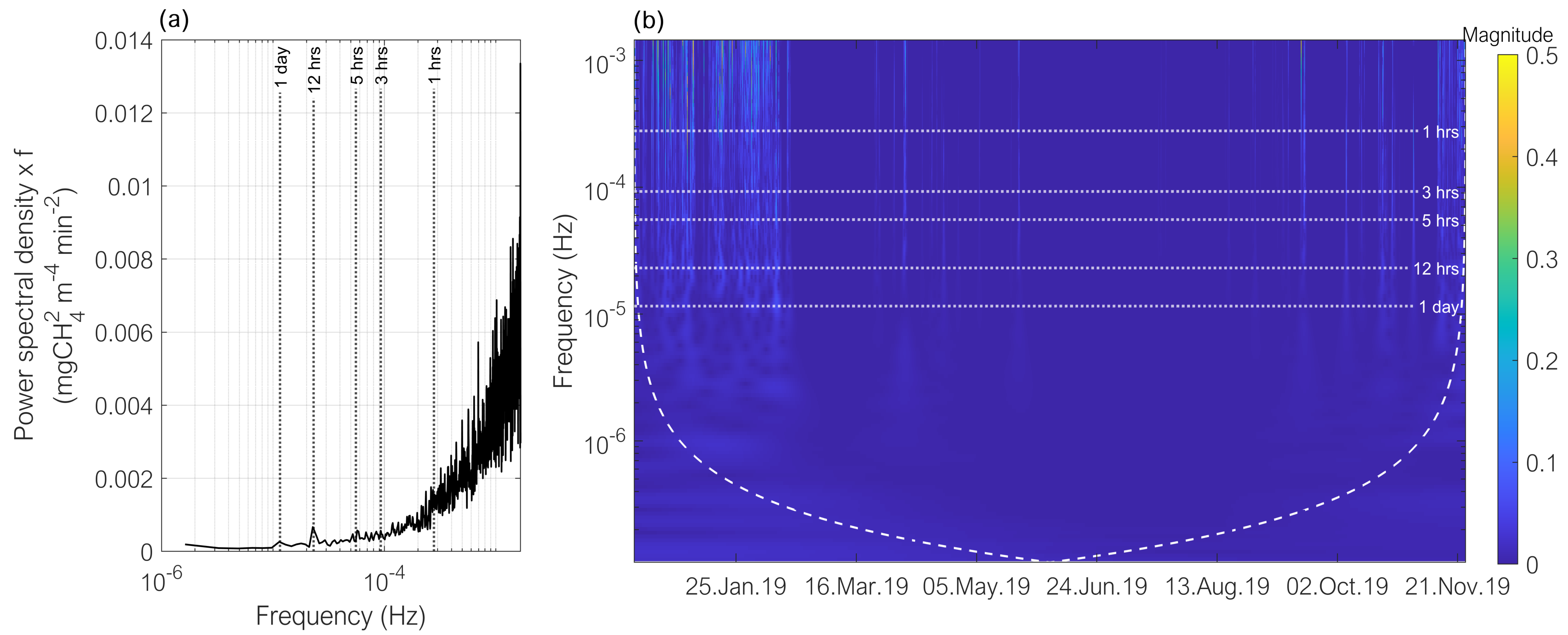

Supplement: S3 Fig — (a) Variance-preserving power spectra of methane ebullition flux at site P1 with a sampling period of 5 min. (b) Wavelet transform of methane ebullition time-series at site P1. The colour represents the absolute value of the continuous wavelet transform in which the yellow colour indicates regions of periodic components and blue regions of low periodic components. The dashed white line shows the cone of influence, in which the areas below the line represent unresolved time scales. For both graphs the period of data used is from December 13th of 2018 to November 24th of 2019. (TIF) [file pone.0298186.s003.tif]

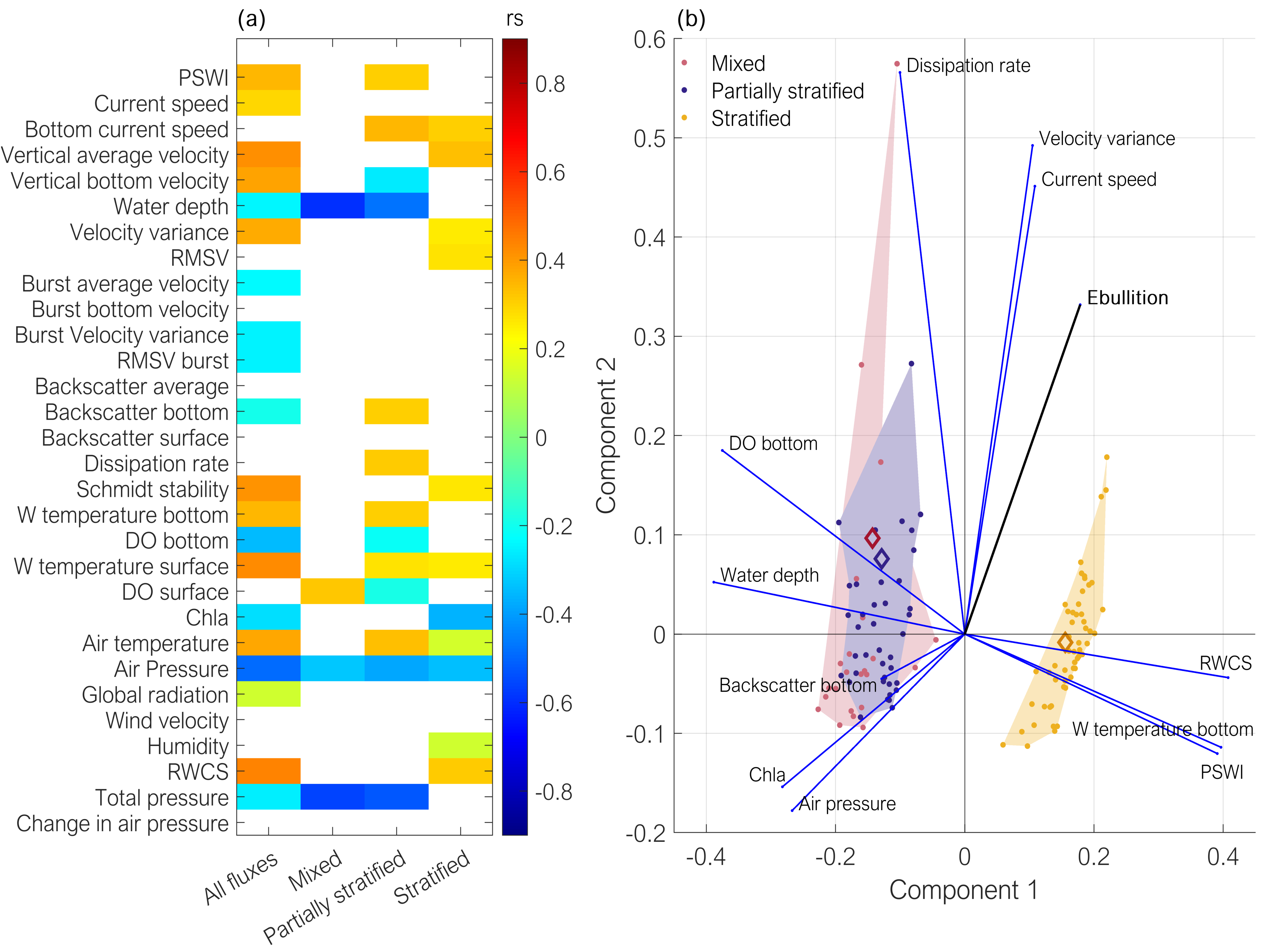

Supplement: S4 Fig — (a) Correlation matrix (Spearman rank correlation) of daily methane ebullition fluxes at location P2 and daily averages of measured environmental parameters. The colour scales with the magnitude of the correlation coefficient (see legend) for a significance level of 0.05 (white boxes refer to no significance correlations with p > 0.05). The first column is the correlation with the complete data set whereas for the 3 other columns the data set was divided according to the prevailing stratification conditions. PSWI refers to the potential methane flux at sediment water interface; RMSV is the Root Mean Square Velocity; W temperature (bottom or surface) is water temperature (near reservoir bed or below the water surface); and Chla is Chlorophyll-a concentration. Total pressure is the sum of hydrostatic and atmospheric pressure. (b) Results of a Principal Component Analysis (PCA) of selected variables with the first two principal components. The dots are data points (daily values) which were grouped based on the stratification conditions by different colours (see legend). Components 1 and 2 explained 47.8% and 15.5% respectively of the data set variability. (TIF) [file pone.0298186.s004.tif]

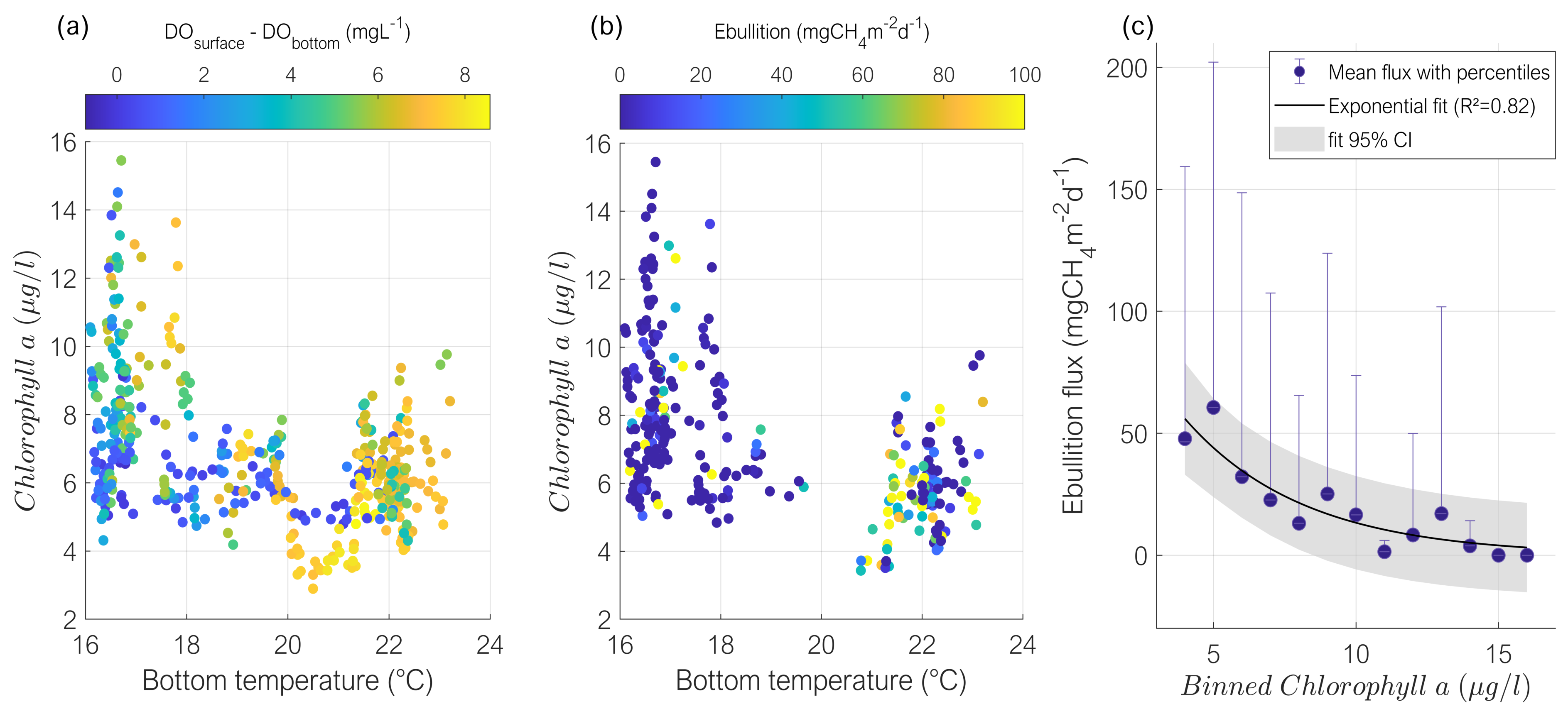

Supplement: S5 Fig — (a) Scatter plot of chlorophyll-a and bottom temperature with the colour scale representing the difference of dissolved oxygen concentrations measured near the surface and near the bottom of the reservoir (see legend). (b) Scatter plot of chlorophyll-a and bottom temperature with the colour scale representing the methane ebullition flux (see legend). (c) Mean methane ebullition flux versus binned chlorophyll-a (by 1 μg L-1). The blue circles show the mean flux for each Chla bin and the error bars are the 10th and 90th percentiles; the solid black line is an exponential fit (y = 145.7 e−0.24 x) to the data and the 95% confidence interval (CI) of the fit is shown by the grey shaded area. All plots are based on daily mean values at monitoring site P2. (TIF) [file pone.0298186.s005.tif]

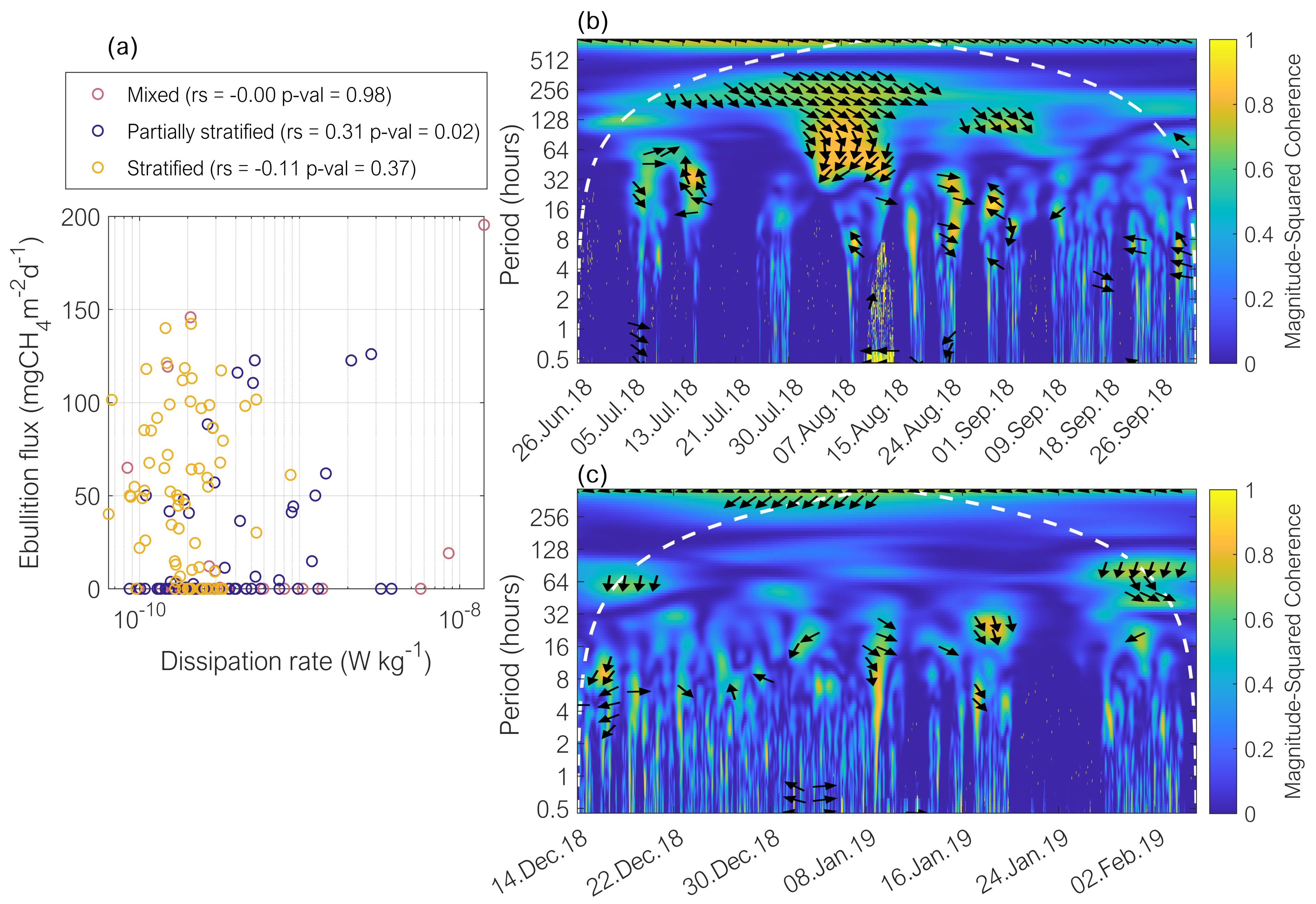

Supplement: S6 Fig — (a) Scatter plot between daily methane ebullition and energy dissipation rates at location P2 grouped by the stratification classification (see colours and legend). Spearman correlation coefficients and significance levels between ebullition and log10-transformed dissipation rates are also shown in the legend. (b) and (c) shows the wavelet coherence analysis between total dissipation rates and ebullition for two intervals of measurements: mixed/partially stratified in (b) and stratified in (c). In both figures, the dashed white lines are the cones of influence, in which the areas above the lines represent time and scales with no dependence in the series. The yellow colour represents regions with high coherence, while blue colour represents lower dependence between the time-series. For both panels the time series are at 10 min time intervals (as the highest time resolution available of dissipation rates are at 10 min time steps). (TIF) [file pone.0298186.s006.tif]
