# Supplementary material for: Exploring the temporal dynamics of methane ebullition in a subtropical freshwater reservoir
Source: PLoS One. 2024 Mar 27;19(3):e0298186. doi: 10.1371/journal.pone.0298186 (PMC10971506; doi:10.1371/journal.pone.0298186)
Supplement: S1 Table — The timescale of each model is provided in the column ‘Variables’. Additional information about the application of the models is provided as footnotes below the table. The model performance on predicting ebullition was evaluated considering the coefficient of determination (R2) of a linear fit between measured and simulated ebullition, the root-mean-square error (RMSE), and the Nash-Sutcliffe efficiency (NSE). The relative error (Relerror in blue color) was calculated between measured and simulated total accumulated flux, in which negative values indicate an overestimation by the model and positive values an underestimation. (PDF) [file pone.0298186.s007.pdf]

**S1 Table. Summary of empirical models from other studies tested for the prediction of ebullition fluxes (y in mL $m^{-2}$ $d^{-1}$ or methane flux in mg $CH_4$ $m^{-2}$ $d^{-1}$) at Passaúna reservoir.** The timescale of each model is provided in the column 'Variables'. Additional information about the application of the models is provided as footnotes below the table. The model performance on predicting ebullition was evaluated considering the coefficient of determination ($R^2$) of a linear fit between measured and simulated ebullition, the root-mean-square error (RMSE), and the Nash-Sutcliffe efficiency (NSE). The relative error ($Rel_{error}$ in blue color) was calculated between measured and simulated total accumulated flux, in which negative values indicate an overestimation by the model and positive values an underestimation.

| Reference | Empirical relationship | Variables | Performance for Passaúna |
|---|---|---|---|
| [1] | $y = -0.00036Tsed^3 + 0.16Tsed^2 - 0.94Tsed + 1.48$ | Temperature binned time series<br>$y$ = mean methane ebullition in mg $CH_4$ $m^{-2}$ $d^{-1}$<br>$Tsed$ = binned (1°C) sediment temperature | $R^2$ = 0.47<br>RMSE = 16.79<br>NSE = 0.23<br>$Rel_{error}$ = −24.3% |
| [1] [2] | $yin_k = W_k + \sum_i WI_j v_i$<br>$y = W + \sum_{i=1}^{3} WS_i \tanh(yin_i)$ | Daily time series<br>$yin_k$ = input value to each neuron<br>$W, WI, WS$ = are weights of the trained neural network<br>$v$ = normalized input variables (change in total static pressure in mH2O, total static pressure in mH2O, and bottom temperature in °C)<br>$y$ = normalized ebullition flux | $R^2$ = 0.03 (0.11)<br>RMSE = 7.40 (18.85)<br>NSE = −6.06 (−44.81)<br>$Rel_{error}$ = 289.5% (770.8%) |
| [2] [3] | Ponds data: $\log_{10}(y) = 0.41 + 0.11Tsed$<br>Lakes data: $\log_{10}(y) = 1.34 + 0.02Tsed$ | Biweekly time-series<br>$y$ = gas ebullition in ml $m^{-2}$ $d^{-1}$<br>$Tsed$ = sediment temperature in °C | Ponds: $R^2$ = 0.17<br>RMSE = 385.11 NSE = −57.68<br>$Rel_{error}$ = −346.9%<br>Lakes: $R^2$ = 0.20<br>RMSE = 48.07 NSE = 0.09<br>$Rel_{error}$ = 39.4% |
| [3] [3] | $\log_{10}(y) = -2.0 + 1.04\log_{10}TP + 0.06Tsed + 0.14(\log_{10}TP - 1.26)(Tsed - 14.65)$ | Biweekly time-series<br>$y$ = methane ebullition in mmol $m^{-2}d^{-1}$<br>$TP$ = total phosphorous (13 µg $L^{-1}$)<br>$Tsed$ = sediment temperature in °C | $R^2$ = 0.19<br>RMSE = 20.2<br>NSE = 0.16<br>$Rel_{error}$ = 30.1% |
| [4] [4] | $y = E_{20} \times \theta^{(T-20)}$ | Daily time series (Temperature binned data)<br>$y$ = methane ebullition in mg $CH_4$ $m^{-2}$ $d^{-1}$ | $R^2$ = 0.01 (0.39)<br>RMSE = 38.73 (18.13)<br>NSE = 0.01 (0.11) |

| | | | |
|---|---|---|---|
| | | $E_{20}$ = methane ebullition at 20°C (36.48 mg $CH_4$ $m^{-2}$ $d^{-1}$)<br>$\theta$ = overall system temperature coefficient (1.22) | $Rel_{error}$ = 5.1% (−17.5%) |
| [5][5] | All sites: $\ln y = -5.11 + 0.37AR1 + 0.30Tsed + 1.14w_s - 0.53\Delta P$ | Weekly time series<br>$y$ = methane ebullition + 0.1 in mg $CH_4$ $m^{-2}$ $d^{-1}$<br>$AR1$ = $\log_e$-transformed methane ebullition from the previous time step (previous week) in ln(in $mgCH_4m^{-2}d^{-1}$)<br>$Tsed$ = sediment temperature in °C<br>$w_s$ = $\log_e$-transformed wind speed in ln(m $s^{-1}$)<br>$\Delta P$ = change in atmospheric pressure in kPa | $R^2$ = 0.25<br>RMSE = 26.53<br>NSE = −0.03<br>$Rel_{error}$ = 62.5% |
| [5][5] | Site T1: $\ln y = -6.46 + 0.32AR1 + 0.39Tsed + 0.39w_s - 0.44\Delta P$ | Weekly time series<br>$y$ = methane ebullition + 0.1 in mg $CH_4$ $m^{-2}$ $d^{-1}$<br>$AR1$ = $\log_e$-transformed methane ebullition from the previous time step (previous week) in ln(in $mgCH_4m^{-2}d^{-1}$)<br>$Tsed$ = sediment temperature in °C<br>$w_s$ = $\log_e$-transformed wind speed in ln(m $s^{-1}$)<br>$\Delta P$ = change in atmospheric pressure in kPa | $R^2$= 0.20<br>RMSE = 26.62<br>NSE= −0.04<br>$Rel_{error}$ = 59.70 |
| [5][5] | Site T2: $\ln y = -2.36 + 0.04AR1 + 0.16Tsed - 0.20Q$ | Weekly time series<br>$y$ = methane ebullition + 0.1 in mg $CH_4$ $m^{-2}$ $d^{-1}$<br>$AR1$ = $\log_e$-transformed methane ebullition from the previous time step (previous week) in ln(in $mgCH_4m^{-2}d^{-1}$)<br>$Tsed$ = sediment temperature in °C<br>$Q$ = $\log_e$-transformed inflow discharge in ln($m^3$ $s^{-1}$) | $R^2$= 0.41<br>RMSE = 37.83<br>NSE= −1.01<br>$Rel_{error}$ = 94.09 |
| [5][5] | Site T3: $\ln y = -2.18 - 0.29AR1 - 0.60Q$ | Weekly time series<br>$y$ = methane ebullition + 0.1 in mg $CH_4$ $m^{-2}$ $d^{-1}$<br>$AR1$ = $\log_e$-transformed methane ebullition from the previous time step (previous week) in ln(in $mgCH_4m^{-2}d^{-1}$) | $R^2$= 0.13<br>RMSE = 55.47<br>NSE= −1.35<br>$Rel_{error}$ = 100.12 |

| | | | |
|---|---|---|---|
| | | $Q$ = log$_e$-transformed inflow discharge in ln(m$^3$ s$^{-1}$) | |
| [5][5] | Site T4: $\ln y = -3.21 + 0.23AR1 + 1.37Phyto$ | Weekly time series<br>$y$ = methane ebullition + 0.1 in mg $CH_4$ m$^{-2}$ d$^{-1}$<br>$AR1$ = log$_e$-transformed methane ebullition from the previous time step (previous week) in ln(in mg$CH_4$m$^{-2}$d$^{-1}$)<br>$Tsed$ = sediment temperature in °C<br>$w_s$ = log$_e$-transformed wind speed in ln(m s$^{-1}$)<br>$\Delta P$ = change in atmospheric pressure in kPa | $R^2$= 0.01<br>RMSE = 41.69<br>NSE= −0.64<br>Rel$_{error}$ = 97.52 |
| [6] [6] | $\ln y = -13.79 + 0.25Tsed + 12.39\varphi + 0.03\,OM$ | Daily time-series<br>$y$ = methane ebullition in mg $CH_4$ m$^{-2}$ d$^{-1}$<br>$Tsed$ = sediment temperature in °C<br>$\varphi$ = sediment porosity (0.9)<br>$OM$ = organic matter (Loss on Ignition 22.9%) | $R^2$ = 0.07<br>RMSE = 37.87<br>NSE = 0.06<br>Rel$_{error}$ = 44.4% |
| [7] [7] | $y = \begin{cases} k\,(P_{th} - P), & P < P_{th} \\ 0, & P \geq P_{th} \end{cases}$<br>$P_{th}(t) = \frac{1}{\tau}\int_{t-\tau}^{t} P(t)dt$ | Daily time series<br>$y$ = methane ebullition in mg $CH_4$ m$^{-2}$ d$^{-1}$<br>$k$ = proportionality constant (94.9 mg $CH_4$ m$^{-2}$ d$^{-1}$ kPa$^{-1}$)<br>$P_{th}$ = time dependent pressure threshold (in kPa)<br>$P$ = Total pressure (in kPa)<br>$\tau$ = time-window over which the threshold is calculated (9 days) | $R^2$ = 0.12<br>RMSE = 52.14<br>NSE = −0.005<br>Rel$_{error}$ = 9.8% |

[1] The model is Multilayer perceptron neural network (MLP) with 3 neurons in the hidden layer. The input variables are normalized to improve model performance. The weights trained by the neural network ($W, WI, WS$) and the coefficients for normalizing the input variables and the ebullition flux are provided by the authors [2]. We tested the model with the input variables normalized as proposed by the author (adopting the provided coefficient for normalization) and by normalizing the data with the minimum and maximum values to obtain the data in the range between 0 and 1. The model performance for the last case is shown in parenthesis.

[2] Biweekly time-series were calculated as the average from the daily time series were, as the authors used measurements with a mean temporal resolution of 15 days.

[3] Mean $TP$ from water samples at P2 of 13 μg $L^{-1}$ [8]

[4] $E_{20}$ = mean ebullition flux from temperature-benned data at 1°C. $\theta$ obtained as $Q_{10} = \theta^{10}$, in which the ecosystem level $Q_{10} = 10^{10\times b}$ with $b$ as the slope of a linear regression between log10(methane Ebullition flux) and (near) sediment temperature. The values in parentheses from the model application refer to the results using binned dataset.

[5] It was adopted weekly time-series of methane ebullition and environmental variables, which were calculated as the average from the daily time series.

[6] $\varphi$ =0.90, porosity assumed as the reservoir sediment is composed mostly by clay and Loss on Ignition (LOI at 550°C) for sediment core at location P2 of 22.9% [9]

[7] The proportionality constant $(k)$ and the time window $(\tau)$ were selected based on the best prediction of methane ebullition.

## References


1. Wik M, Thornton BF, Bastviken D, MacIntyre S, Varner RK, Crill PM. Energy input is primary controller of methane bubbling in subarctic lakes. Geophys Res Lett. 2014;41: 555–560. doi:10.1002/2013GL058510
2. Deshmukh C, Serça D, Delon C, Tardif R, Demarty M, Jarnot C, et al. Physical controls on CH4 emissions from a newly flooded subtropical freshwater hydroelectric reservoir: Nam Theun 2. Biogeosciences. 2014;11: 4251–4269. doi:10.5194/bg-11-4251-2014
3. DelSontro TS, Boutet L, St-Pierre A, del Giorgio PA, Prairie YT. Methane ebullition and diffusion from northern ponds and lakes regulated by the interaction between temperature and system productivity. Limnol Oceanogr. 2016;61: S62–S77. doi:10.1002/lno.10335
4. Aben RCH, Barros N, Van Donk E, Frenken T, Hilt S, Kazanjian G, et al. Cross continental increase in methane ebullition under climate change. Nat Commun. 2017;8: 1–8. doi:10.1038/s41467-017-01535-y
5. McClure RP, Lofton ME, Chen S, Krueger KM, Little JC, Carey CC. The Magnitude and Drivers of Methane Ebullition and Diffusion Vary on a Longitudinal Gradient in a Small Freshwater Reservoir. J Geophys Res Biogeosciences. 2020;125: 0–18. doi:10.1029/2019JG005205
6. Praetzel LSE, Schmiedeskamp M, Knorr K. Temperature and sediment properties drive spatiotemporal variability of methane ebullition in a small

and shallow temperate lake. Limnol Oceanogr. 2021;66: 2598–2610. doi:10.1002/lno.11775

7. Zhao K, Tedford EW, Lawrence GA. Ebullition Regulated by Pressure Variations in a Boreal Pit Lake. Front Earth Sci. 2022;10: 1–7. doi:10.3389/feart.2022.850652

8. Gurski LKK. Optimal resolutions for modeling and monitoring the water quality dynamics of Passaúna's reservoir. Karlsruhe Institute of Technology (KIT) and Federal University of Paraná (UFPR). 2022.

9. Sotiri K, Hilgert S, Mannich M, Bleninger T, Fuchs S. Implementation of comparative detection approaches for the accurate assessment of sediment thickness and sediment volume in the Passaúna Reservoir. J Environ Manage. 2021;287: 112298. doi:10.1016/j.jenvman.2021.112298
