# Supplementary material for: Exploring the temporal dynamics of methane ebullition in a subtropical freshwater reservoir
Source: PLoS One. 2024 Mar 27;19(3):e0298186. doi: 10.1371/journal.pone.0298186 (PMC10971506; doi:10.1371/journal.pone.0298186)
Supplement: S2 Table — The timescale of each model is provided in the column ‘Variables’. Additional information about the application of the models are provided as footnotes below the table. The model performance in predicting ebullition was evaluated considering the coefficient of determination (R2) of a linear fit between measured and simulated ebullition, the root-mean-square error (RMSE), and the Nash-Sutcliffe efficiency (NSE). The relative error (Relerror) was calculated between measured and simulated total accumulated flux, in which negative values indicate an overestimation by the model and positive values an underestimation. (PDF) [file pone.0298186.s008.pdf]

S2 Table. Empirical models tested for the prediction of ebullition flux (y in mL m^{-2}d^{-1} or methane flux in mg CH_4 m^{-2} d^{-1}) based on data from Passaúna reservoir. The timescale of each model is provided in the column 'Variables'. Additional information about the application of the models are provided as footnotes below the table. The model performance in predicting ebullition was evaluated considering the coefficient of determination (R^2) of a linear fit between measured and simulated ebullition, the root-mean-square error (RMSE), and the Nash-Sutcliffe efficiency (NSE). The relative error (Rel_{error}) was calculated between measured and simulated total accumulated flux, in which negative values indicate an overestimation by the model and positive values an underestimation.

Method	Empirical equation	Variables	Performance for Passaúna
Models from Table S1 with coefficients recalculated for Passaúna reservoir			
Polynomial fit	$y = -0.024Tsed^3 + 0.006Tsed^2 + 36.57Tsed - 496.7$	Temperature binned data<br>y = mean methane ebullition in mg CH_4 m^{-2} d^{-1}<br>$Tsed$ = binned (1°C) sediment temperature	R^2 = 0.62<br>RMSE = 11.79<br>NSE = 0.62<br>Rel_{error} = 2.06x10^{-13}%
Linear fit	$\log_{10}(y) = -1.78 + 0.15Tsed$	Biweekly time series<br>y = mean methane ebullition + 0.1 in mg CH_4 m^{-2} d^{-1}<br>$Tsed$ = sediment temperature in °C	R^2 = 0.16<br>RMSE = 22.73<br>NSE = −0.07<br>Rel_{error} = 59.1%
Linear fit	$\log_{10}(y) = -3.50 + 0.19Tsed$	Daily time series<br>y = methane ebullition + 0.1 in mg CH_4 m^{-2} d^{-1}<br>$Tsed$ = sediment temperature in °C	R^2 = 0.06<br>RMSE = 43.86<br>NSE = −0.26<br>Rel_{error} = 93.1%
Multiple linear regression	$\ln y = -8.48 + 0.20AR1 + 0.46Tsed + 2.92w_s - 0.24\Delta P$	Weekly time series<br>y = methane ebullition + 0.1 in mg CH_4 m^{-2} d^{-1}<br>$AR1$ = $\log_e$-transformed methane ebullition +0.1 from the previous time step (previous week) in ln(in mgCH_4m^{-2}d^{-1})<br>$Tsed$ = sediment temperature in °C<br>w_s = $\log_e$-transformed wind speed in ln(m s^{-1})<br>ΔP = change in atmospheric pressure in kPa	R^2 = 0.24<br>RMSE = 25.74<br>NSE = 0.03<br>Rel_{error} = 59.0%
Multiple linear regression	$\ln y = -3.64 + 0.61AR1 + 0.19Tsed + 0.33w_s + 2.88\Delta P$	Daily time series<br>y = methane ebullition + 0.1 in mg CH_4 m^{-2} d^{-1}	R^2 = 0.31<br>RMSE = 38.47<br>NSE = 0.02

		$AR1$ = $\log_e$-transformed methane ebullition +0.1 from the previous time step (previous week) in ln(in mg CH_4 m^{-2} d^{-1})<br>$Tsed$ = sediment temperature in °C<br>w_s = $\log_e$-transformed wind speed in ln(m s^{-1})<br>ΔP = change in atmospheric pressure in kPa	Rel_{error} = 74.4%
[1] Artificial neural network (ANN)	$yin_k = b_{in,k} + \sum_i x_i w_{ij}$<br>$yout_k = \frac{2}{1+e^{-2\times yin_k}} - 1$<br>$y = b_{out} + \sum_i yout_i v_i$	Daily time series<br>yin_k = values of each neuron in the hidden layer<br>b_{in}, b_{out} = are bias of hidden neurons and of output layer<br>w, v = are weights of the trained neural network (hidden layer and output neuron)<br>x = normalized input variables (change in total static pressure in Pa, total static pressure in Pa, and bottom temperature in °C)<br>$yout_k$ = output value of the sigmoid transfer function<br>y = normalized ebullition flux	R^2 = 0.32<br>RMSE = 0.31<br>NSE = 0.31<br>Rel_{error} = 4.4%
New models developed based on Passaúna dataset			
[2] Stepwise regression	$y = 1.79\times10^5 + 1976.7v_{bottom} - 194.21P_{air} - 0.85P_{total} + 9.26\times10^{-4}(P_{air}\times P_{total})$	Daily time series<br>y = methane ebullition in $mgCH_4m^{-2}d^{-1}$<br>v_{bottom} = bottom current speed in m s^{-1}<br>P_{air} = atmospheric pressure<br>P_{total} = total pressure (hydrostatic + atmospheric)	R^2 = 0.10<br>RMSE = 51.91<br>NSE = 0.004<br>Rel_{error} = −2.31%
[2] Stepwise regression (log-transformed)	$\log_{10}(y) = 1033.8 + 0.78\,v_{var} - 3.42T_{sed} - 206.75P_{air} - 77.81P_{total}$	Daily time series<br>y = methane ebullition + 1 in $mgCH_4m^{-2}d^{-1}$<br>v_{var} = $\log_{10}$-transformed velocity variance<br>T_{sed} = $\log_{10}$-transformed sediment temperature<br>P_{air} = $\log_{10}$-transformed atmospheric pressure<br>P_{total} = $\log_{10}$-transformed total pressure (hydrostatic + atmospheric)	R^2 = 0.26<br>RMSE = 41.94<br>NSE = 0.11<br>Rel_{error} = 48.11%

[3] Multiple linear regression	$y = 5.89 \times 10^3 + 1.58 \times 10^3 v_{bottom} + 1.18 \times 10^5 v_{var} - 2.45T_{sed} - 1.07DO_{bot} - 5.08P_{air} + 0.14w_s + 0.005RWCS - 0.006P_{total}$	Daily time series<br>y = methane ebullition in $mgCH_4m^{-2}d^{-1}$<br>v_{bottom} = bottom current in m s^{-1}<br>v_{var} = velocity variance at bottom in $m^{-2}s^{-2}$<br>T_{sed} = sediment temperature in °C<br>DO_{bot} = dissolved oxygen concentrations in mg L^{-1}<br>P_{air} = air pressure in mbar<br>w_s = wind speed in m s^{-1}<br>$RWCS$= relative water column stability<br>P_{total} = total pressure (hydrostatic + atmospheric) in Pa	R^2 = 0.37<br>RMSE = 35.25<br>NSE = 0.37<br>Rel_{error} = −1.89%
[3] Multiple linear regression with AR1 term	$y = 4.46 \times 10^3 + 1.54 \times 10^3 v_{bottom} + 1.39 \times 10^5 v_{var} - 0.99T_{sed} - 1.54DO_{bot} - 3.78P_{air} - 2.26w_s - 0.06RWCS - 0.005P_{total} + 0.20AR1$	Daily time series<br>y = methane ebullition in $mgCH_4m^{-2}d^{-1}$<br>v_{bottom} = bottom current in m s^{-1}<br>v_{var} = velocity variance at bottom in $m^{-2}s^{-2}$<br>T_{sed} = sediment temperature in °C<br>DO_{bot} = dissolved oxygen concentrations in mg L^{-1}<br>P_{air} = air pressure in mbar<br>w_s = wind speed in m s^{-1}<br>$RWCS$= relative water column stability<br>P_{total} = total pressure (hydrostatic + atmospheric) in Pa<br>$AR1$ = = log_e-transformed methane ebullition +1 from the previous time step (previous day)	R^2 = 0.39<br>RMSE = 34.60<br>NSE = 0.39<br>Rel_{error} = −1.27%
[3] Multiple linear regression with AR1 term (log_{10}-transformed)	$\log_{10}(y) = 608.00 + 0.48v_{bottom} + 0.73v_{var} + 0.045\varepsilon - 4.45T_{sed} - 0.26DO_{bot} - 117.37P_{air} + 0.14w_s - 0.06RWCS - 47.05P_{total} + 0.36AR1$	Daily time series<br>y = methane ebullition + 1 in $mgCH_4m^{-2}d^{-1}$<br>v_{bottom} = log_{10}-transformed bottom current in m s^{-1}<br>v_{var} = log_{10}-transformed velocity variance at bottom in $m^{-2}s^{-2}$<br>ε = log_{10}-transformed dissipation rates near bottom in W kg^{-1}<br>T_{sed} = log_{10}-transformed sediment temperature in °C	R^2 = 0.28<br>RMSE = 40.44<br>NSE = 0.17<br>Rel_{error} = 40.8%

		DO_{bot} = log_{10}-transformed dissolved oxygen concentrations in mg L^{-1}<br>P_{air} = log_{10}-transformed air pressure in mbar<br>w_s = log_{10}-transformed wind speed in m s^{-1}<br>$RWCS$= log_{10}-transformed relative water column stability<br>P_{total} = log_{10}-transformed total pressure (hydrostatic + atmospheric) in Pa<br>$AR1$ = = log_e-transformed methane ebullition +1 from the previous time step (previous day)	
[4] Generalized additive model (GAM) for regression	$y \sim N(\mu, \sigma^2)$<br>$g(\mu) = 45.18 + \sum_i f_i(x_i)$	Daily time series<br>y = methane ebullition in $mgCH_4m^{-2}d^{-1}$, assuming a normal distribution with mean μ and standard deviation σ<br>$g(\mu) = \mu$ = identity link function<br>$f_i(x_i)$ = is a univariate shape function for the *ith* predictor	R^2 = 0.70<br>RMSE = 27.34<br>NSE = 0.69<br>Rel_{error} = −0.28%<br>Predicted-R^2 = < 0
[5] Artificial Neural Network (ANN) (9 input variables)	$yin_k = b_{in,k} + \sum_i x_i w_{ij}$<br>$yout_k = \frac{2}{1 + e^{-2 \times yin_k}} - 1$<br>$y = b_{out} + \sum_i yout_i v_i$	Daily time series<br>yin_k = values of each neuron in the hidden layer<br>b_{in}, b_{out} = are bias of hidden neurons and of output layer<br>w, v = are weights of the trained neural network (hidden layer and output neuron)<br>x = normalized input variables (see bottom notes)<br>$yout_k$ = output value of the sigmoid transfer function<br>y = normalized ebullition flux	R^2 = 0.41<br>RMSE = 0.29<br>NSE = 0.41<br>Rel_{error} = 2.6%
Testing best model for prediction in different timescales			
[6] Generalized additive model (GAM) for regression	$y \sim N(\mu, \sigma^2)$<br>$g(\mu) = 0.03 + \sum_i f_i(x_i)$	10-minutes time-series<br>y = methane ebullition in $mgCH_4m^{-2}hr^{-1}$, assuming a normal distribution with mean μ and standard deviation σ<br>$g(\mu) = \mu$ = identity link function	R^2 = 0.05<br>RMSE = 0.11<br>NSE = 0.049<br>Rel_{error} = −1.2%<br>Predicted-R^2 = < 0

		$f_i(x_i)$ = is a univariate shape function for the *ith* predictor	
[4] Generalized additive model (GAM) for regression	$y \sim N(\mu, \sigma^2)$<br>$g(\mu) = 1.85 + \sum_i f_i(x_i)$	Hourly time-series<br>y = methane ebullition in $mgCH_4m^{-2}hr^{-1}$, assuming a normal distribution with mean μ and standard deviation σ<br>$g(\mu) = \mu$ = identity link function<br>$f_i(x_i)$ = is a univariate shape function for the *ith* predictor	R^2 = 0.19<br>RMSE = 3.22<br>NSE = 0.19<br>Rel_{error} = −1.24%<br>Predicted-R^2 = < 0
[4] Generalized additive model (GAM) for regression	$y \sim N(\mu, \sigma^2)$<br>$g(\mu) = 37.68 + \sum_i f_i(x_i)$	Weekly time-series<br>y = methane ebullition in $mgCH_4m^{-2}d^{-1}$, assuming a normal distribution with mean μ and standard deviation σ<br>$g(\mu) = \mu$ = identity link function<br>$f_i(x_i)$ = is a univariate shape function for the *ith* predictor	R^2 = 0.96<br>RMSE = 6.4<br>NSE = 0.96<br>Rel_{error} = 0.01%<br>Predicted-R^2 = < 0

[1] ANN: output layer with three inputs (normalized to range 0 and 1); one hidden layer with five neurons and a sigmoid transfer function; and one output layer as the normalized daily methane ebullition flux (fluxes normalized to range between -1 and +1). The ANN was trained using the 'Neural Network Fitting' toolbox at MatLab 2023a. The toolbox uses the Levenberg—Marquardt training algorithm and the data points (n = 149) are randomly divided into training set (70%), validation (15%), and testing (15%). Tha validation dataset is used by the algorithm to stop training the ANN when the error in predicting the validation data continuously increases for more than 6 (Default) Epochs.

[2] For the Stepwise regression nine variables were potential ebullition predictors: bottom current, velocity variance at the bottom, dissipation rate near the bottom, sediment temperature, dissolved oxygen concentrations near bottom, atmospheric pressure, wind speed, relative water column stability (RWCS) and total pressure. Here, the predictors are included sequentially in the model and accepted if a p-value criterion is met for a significance level of 5%. Interactions among the input variables are also tested as potential predictors.

[3] For the multiple linear regression the same nine variables from the Stepwise regression were considered as potential predictor (adding ebullition with time-lag of 1 in the last 2 cases of the multiple linear regression). Here the main difference to the Stepwise linear regression is that interactions among the input variables are not considered as potential predictors.

[4] The model was created using the *fitrgam* function in MatLab. In the model methane ebullition is explained using a sum of univariate shape functions of predictors. The input predictors were the nine variables used on the Stepwise regression. The model can handle non-linear interactions between ebullition and each predictor. The shape function for each predictor in created using a boosted tree. The model was also tested with log-transformed variables, however no improvement was achieved. The predicted-R^2 was estimated as an indication for model overfitting. It was calculated applying the Leave One Out Cross-Validation method as $predR^2 = 1 - \frac{PRESS}{SSTO}$, where $PRESS$ is the prediction sum of squares calculated by omitting each observation individually and $SSTO$ is the total sum of squares considering all datapoints.

[5] ANN: output layer with the same nine inputs variables adopted for Stepwise regression (normalized to range 0 and 1); one hidden layer with twenty neurons and a sigmoid transfer function; and one output layer as the normalized daily methane ebullition flux (fluxes normalized to range between -1 and +1). The ANN was trained using the 'Neural Network Fitting' toolbox at MatLab 2023a. We adopted the Bayesian regularization training algorithm and the data points (n = 149) are randomly divided into training set (65%), validation (15%), and testing (20%). Different architectures of the ANN was tested, here we present the configuration with the best results. Tha validation dataset is used by the algorithm to stop training the ANN when the error in predicting the validation data continuously increases for more than 6 (Default) Epochs.

[6] For teste the GAM model with the 10 minutes timestep time series, the velocity variance wasn't included as a predictor, because it was only available for time intervals ≥ 1 hr. Therefore, eight predictors were considered: bottom current, dissipation rate near the bottom, sediment temperature, dissolved oxygen concentrations near bottom, atmospheric pressure, wind speed, relative water column stability (RWCS) and total pressure.
